# Structural characterization of plum pox virus by cryo-electron microscopy

**DOI:** 10.1007/s00705-025-06473-5

**Published:** 2025-12-01

**Authors:** Diane Marie Valérie Jeanne Bonnet, Antonio Chaves-Sanjuan, Nicoletta Contaldo, Angelo De Stradis, Rosanna Caliandro, Angelantonio Minafra, Filippo Geuna

**Affiliations:** 1 Unitech NOLIMITS, Imaging Facility, Università degli Studi di Milano, Milan, Italy; 2 Department of Biosciences, Università degli Studi di Milano, Milan, Italy; 3 Institute for Sustainable Plant Protection (IPSP) - Consiglio Nazionale delle Ricerche (CNR), Bari, Italy; 4 Department of Agricultural and Environmental Sciences (DISAA), Università degli Studi di Milano, Milan, Italy

**Keywords:** Potyvirus, Structure prediction, Mass spectrometry, AlphaFold, Post-translational modification

## Abstract

Plum pox virus (PPV), a significant member of the genus *Potyvirus*, represents a global agricultural challenge, causing significant economic losses and threatening fruit farming due to its easy transmission to most *Prunus* species. Here, we present the high-resolution structural characterization of PPV using cryo-electron microscopy (cryo-EM). The reconstructed structure at 2.9 Å reveals a filamentous virion with a helical assembly formed by the coat protein (CP), which encapsidates a single-stranded RNA (ssRNA) genome. The structure of the CP core shows remarkable conservation with other potyviruses, with an RNA binding site and inter-subunit interactions mediated in part by the N-terminal arm, which is confirmed here to have a disordered structure. Mass spectrometry analysis identified numerous post-translational modifications, mostly phosphorylation, primarily in the flexible N-terminal region. *In silico* predictions revealed intrinsically disordered regions, which is compatible with the amyloidogenic properties of the CP. These results provide new insights into the architecture and assembly of PPV, offering a basis for future studies and, possibly, antiviral strategies.

**Supplementary information:**

The online version contains supplementary material available at 10.1007/s00705-025-06473-5.

## Introduction

The genus *Potyvirus* (family *Potyviridae*) represents the largest known group of plant RNA viruses, encompassing over 180 species and constituting approximately 15% of all identified plant viruses [1, 2]. These viruses are significant agricultural pathogens, causing substantial economic losses worldwide by affecting crop yield and quality [1]. In some developing countries, potyviruses pose a threat to food security due to the devastating diseases they cause in tropical and subtropical areas [3]. In particular, plum pox virus (PPV, *Potyvirus plumpoxi*) represents a global agricultural challenge due to the ease of transmission of its different strains to most of the *Prunus* species [4].

The impact on fruticulture is thus significant in terms of fruit loss and because it necessitates the eradication of infected trees, further increasing containment costs. The economic losses have been estimated at over €2.4 billion in the last 28 years [4]. PPV has several strains, including D, M, EA, C, Rec, W, T, CR, and An, each with unique genetic and biological properties [1]. These strains differ in their host range, aphid transmissibility, symptom severity, and geographic distribution [5]. Strain M is particularly important because of its high aggressiveness and efficient aphid transmission [6]. It spreads rapidly in orchards, especially affecting plum, peach, and apricot trees [7].

Members of the family *Potyviridae* typically possess a single-stranded, positive-sense RNA genome, although some exceptions exist [1]. Within this family, the genus *Potyvirus* is the most extensive, with species characterized by a monopartite genome of around 10 kb, (e.g., 9.7 kb in most PPV isolates), consisting of the main large open reading frame (ORF) of about 9.4 kb flanked by 5' and 3' noncoding regions (NCRs). This genome is translated into a large polyprotein, which is subsequently processed by three different virus-encoded viral proteases into mature proteins [7, 8]. Other viral proteins have been identified to partner with CPs to promote virion assembly [8].

A critical aspect of the potyvirus life cycle is the assembly of new virions, a process involving the specific packaging of the genomic RNA by the coat protein (CP) [8]. Furthermore, the CP itself is involved in multiple functions beyond encapsidation, highlighting the need for tight regulation of its activity and availability throughout the infection cycle [9, 10]. Premature or non-specific encapsidation can hinder viral RNA translation and replication [11]. This regulation involves complex mechanisms, including post-translational modifications (PTMs) such as phosphorylation and O-GlcNAcylation, as well as interactions with host factors [12–15].

To gain deeper insights into the structure and assembly of these virions, high-resolution structural techniques are essential. Cryo-electron microscopy (cryo-EM) has emerged as a powerful tool in structural biology, enabling the visualization of biological macromolecules in their near-native state [16, 17]. It has significantly advanced the study of plant viruses, providing unprecedented detail, as exemplified by the high-resolution structure determination of potato virus X (PVX), a flexible, filamentous virus, and other viruses affecting crops of agronomic interest [18–24]. Such structural information is invaluable for understanding virus assembly and function, and for designing structure-based antiviral strategies.

In this study, we used cryo-EM to determine the structure of PPV, providing important details about its virion architecture and contributing to a better understanding of potyvirus assembly and biology.

## Materials and methods

### Purification and CP sequencing of the PPV-M isolate

PPV (M strain, isolate GR0046) was obtained from an infected peach (*Prunus persica*) tree in Greece and characterized within the framework of a SHARCO EU-funded project (‘Sharka Containment in view of EU expansion’, 7th FP 2006–2011). The virus was maintained in *Nicotiana benthamiana* plants through mechanical inoculation, and virus particles were partially purified as described by Berger et al. [53]. At 21 days post-inoculation, ca. 100 g of leaves from infected plants were homogenized in 3 volumes of Tris-HCl buffer (0.1 M, pH 8.0) containing 0.3% sodium metabisulfite and 0.01% Triton X-100. The filtered slurry was mixed with 1/3 volume of chloroform-butanol (1:1 v/v) by stirring on ice for 30 minutes. To the supernatant obtained after a low-speed centrifugation, 8% polyethylene glycol (PEG 8,000) and 1% NaCl were added to precipitate the virus particles for 1 h on ice. The suspension was then centrifuged, and the pellet was resuspended overnight at 4 °C with a small volume of 10 mM Tris-HCl buffer (pH 8.0). After a short centrifugation at low speed to remove residual plant material, high-speed centrifugation on a 30% sucrose cushion was done at 36,000 rpm for 2 h. The final pellet was then resuspended in 0.5 ml of 10 mM Tris-HCl buffer (pH 8.0), and the optical density was determined using a NanoDrop spectrophotometer (Thermo Fisher Scientific). By assuming an extinction coefficient at 260 nm of about 3.0 for PPV particles, the final virus yield was estimated to be 0.85 mg/ml.

To determine the size of the CP in partially purified particles, SDS-PAGE and Western blot analysis was done using 100 mg of healthy or PPV-infected *N. benthamiana* leaf tissue extracted in PBS buffer. The gel was blotted and probed with alkaline phosphatase (AP)-conjugated polyclonal antibodies (Agritest, Italy) for colorimetric detection.

The quality of the purified PPV suspension was assessed by TEM as described by Milne [54]. A small drop (20 µl) of purified suspension was applied to a carbon-coated copper/rhodium 400-mesh grid (TAAB Laboratories Equipment Ltd, Aldermaston, Berks, GB). The coated grid was floated for 2 min on the sample drop and rinsed with 200 µl of double-distilled water. Negative staining was performed with 200 µl of a 2% (w/v) uranyl acetate solution (UA-Zero EM Stain, Agar Scientific, UK). The specimen was transferred to a Philips Morgagni 282D transmission electron microscope operating at 80 kV. Electron micrographs of negatively stained samples were photographed using Kodak 4489 film (Kodak Company, New York, USA).

Viral RNA was purified from partially purified particles by extraction with TRIzol-chloroform and ethanol precipitation. A 3’-terminal fragment of the PPV genome (*ca*. 1,400 bp) containing the coat protein (CP) sequence was amplified from reverse-transcribed cDNA using the primers P1F/P3R [55]. The fragment was TA-ligated into the plasmid pSCa (Stratagene) and cloned in *E. coli*. Clone #7 was then selected for sequencing of the CP gene.

### Cryo-EM methods

Sample vitrification was carried out using a Mark IV Vitrobot (Thermo Fisher Scientific). Three µl of PPV in dispersion at 3.3 mg/ml was applied to a Quantifoil R 2/1 Cu 300-mesh grid that had been glow-discharged at 30 mA for 30 seconds in a GloQube (Quorum Technologies). Immediately after application of the sample, the grid was blotted in a chamber at 4ºC and 100% humidity and then plunge-frozen in liquid ethane.

Vitrified grids were transferred to a Talos Arctica cryogenic transmission electron microscope (TEM) (Thermo Fisher Scientific) operated at 200 kV and equipped with a Falcon3 detector (Thermo Fisher Scientific). Images were acquired at a nominal magnification of 120,000x, corresponding to a pixel size of 0.889 Å/pixel with a defocus range of −0.5 µm to −2.3 µm and with a dose of 1e-/Å^2^/fraction and a total of 40 fractions.

The cryo-EM experiments were carried out at the Unitech NOLIMITS center of the University of Milan.

### Data processing methods

The best movies (without the presence of artifacts, crystalline ice, severe astigmatism, or obvious drift) were used to process the data. A total of 2508 movies were imported into RELION3.1 [56, 57]. Motion correction and dose weighting were carried out using MOTIONCORR2 [58]. CTF estimation was performed using CTFFIND4 [59]. Filament coordinates were picked manually start-to-end and extracted in 344 pixel boxes with an interbox distance of 10%, producing 406,769 initial segments. The segments were imported into CRYOSPARC 4.6.2 [60] and subjected to helical refinement using the volume of PVY (EMD-0297 [23]) low-pass filtered at a resolution of 40 Å as a reference. A subsequent local CTF refinement and helical refinement produced the final reconstruction at 2.9 Å resolution, with the helical parameters ΔΖ = 4.08 Å and ΔΦ= −40.89° (Supplementary Fig. S1 and Supplementary Table S1).

### High-resolution mass spectrometry analysis (nLC-HRMS)

On the entire sample volume available (150 µl), an enzymatic digestion in solution was performed according to the UNITECH OMICs (University of Milano, Italy) platform protocol, which involves, in order, treatment with:

- DTT (final concentration 5 mM); 55 °C for 30 min;
- isoamyl alcohol (final concentration 15 mM); in the dark for 20 min;
- trypsin (0.2 µg/µl); 37 °C overnight;
- trifluoroacetic acid (100%) and subsequent purification using desalting columns (Pierce Peptide Desalting Spin Columns).

The obtained eluate was dried, dissolved in 200 µl of binding/equilibration buffer, and subjected to a phosphopeptide enrichment protocol using a High-Select Fe-NTA Phosphopeptide Enrichment Kit (Thermo Scientific, catalog no. A32992) to obtain eluate for phosphopeptide analysis and flowthrough (FT) for total proteome analysis. Both samples were dried completely in a SpeedVac concentrator (Thermo Fisher Scientific) before instrumental analysis.

The sample for phosphopeptide analysis was dissolved in 10 µl of 0.1% formic acid in water, and 2 µl of the dissolved sample was injected into the column. The sample for total proteome analysis (FT) was dissolved in 100 µl of 0.1% formic acid in water, and 4 µl of the dissolved sample was injected into the column.

All samples were analyzed using a Dionex UltiMate 3000 Nano LC System (Sunnyvale CA, USA) connected to an Orbitrap Fusion Tribrid Mass Spectrometer (Thermo Scientific, Bremen, Germany) equipped with a nano-electrospray ion source. Peptide mixtures were pre-concentrated on a PepMap 100 C18 column (300 µm × 5 mm; Thermo Scientific) and separated on an EASY-Spray ES900 column (15 cm × 75 µm ID) packed with Thermo Scientific Acclaim PepMap RSLC C18 (3 µm, 100 Å) using mobile phase A (0.1% formic acid in water) and mobile phase B (0.1% formic acid in acetonitrile [20/80, v/v]) at a flow rate of 0.300 µl/min. The elution program is summarized in Supplementary Table S2.

The temperature was set to 35 °C, and samples were injected in duplicate. The sample injection volume was 2 or 4 µl. One blank was run between samples to prevent sample carryover. MS spectra were collected over an m/z range of 375-1,500 Da at a resolution of 120,000, operating in data-dependent mode with a cycle time of 3 seconds between master scans. HCD was performed with the collision energy (CE) set at 30% for phosphopeptide, and EThcD was performed with the CE set at 15% for HexNac modifications. The polarity was positive.

All samples were processed using Proteome Discoverer 2.5 software, setting the search database to Plum Pox Virus SK 68 (sp_tr_incl_isoforms TaxID = 103927_and_subtaxonomies) (v2024-07–24) and trypsin as the digestion enzyme (Max. Missed Cleavage Sites: 2). A list of the modifications tested is available in Supplementary File 1.

### Calculation of amyloidogenic properties

The predicted amyloidogenic properties of the coat protein were predicted using AMYLPRED2 software (http://thalis.biol.uoa.gr/AMYLPRED2/) [61], which employs a set of different methods that have been developed to predict features related to the formation of amyloid fibrils. The consensus of these methods is defined as the hit overlap of at least n/2 (rounded down) out of n selected methods.

### Calculation of protein disorder

We used the protein disorder predictor Espritz [62, 63] to identify likely disordered residues and segments. Espritz makes reference to disorder annotations based on nuclear magnetic resonance (NMR) structures, X-ray crystal structures, and experimental annotations from the DisProt database and server [64, 65].

### Protein folding prediction

Protein fold similarity searches and predictions were carried out using the AlphaFold server (version beta-20231127) and database [66, 67]. Prediction accuracy was assessed using the following metrics: global distance test (GDT), template modeling score (TM-score), root mean square deviation (RMSD), and local distance difference test (LDDT) [68, 69]. Results were visualized using Chimera [70].

## Results

### Virus purification and CP sequence

The PPV strain M isolate was grown in the herbaceous host *Nicotiana benthamiana* and, following purification by ultracentrifugation, was used for a preliminary electron microscopy (EM) analysis to determine the amount of virus in the sample and its degree of purity.

**Fig. 1:**
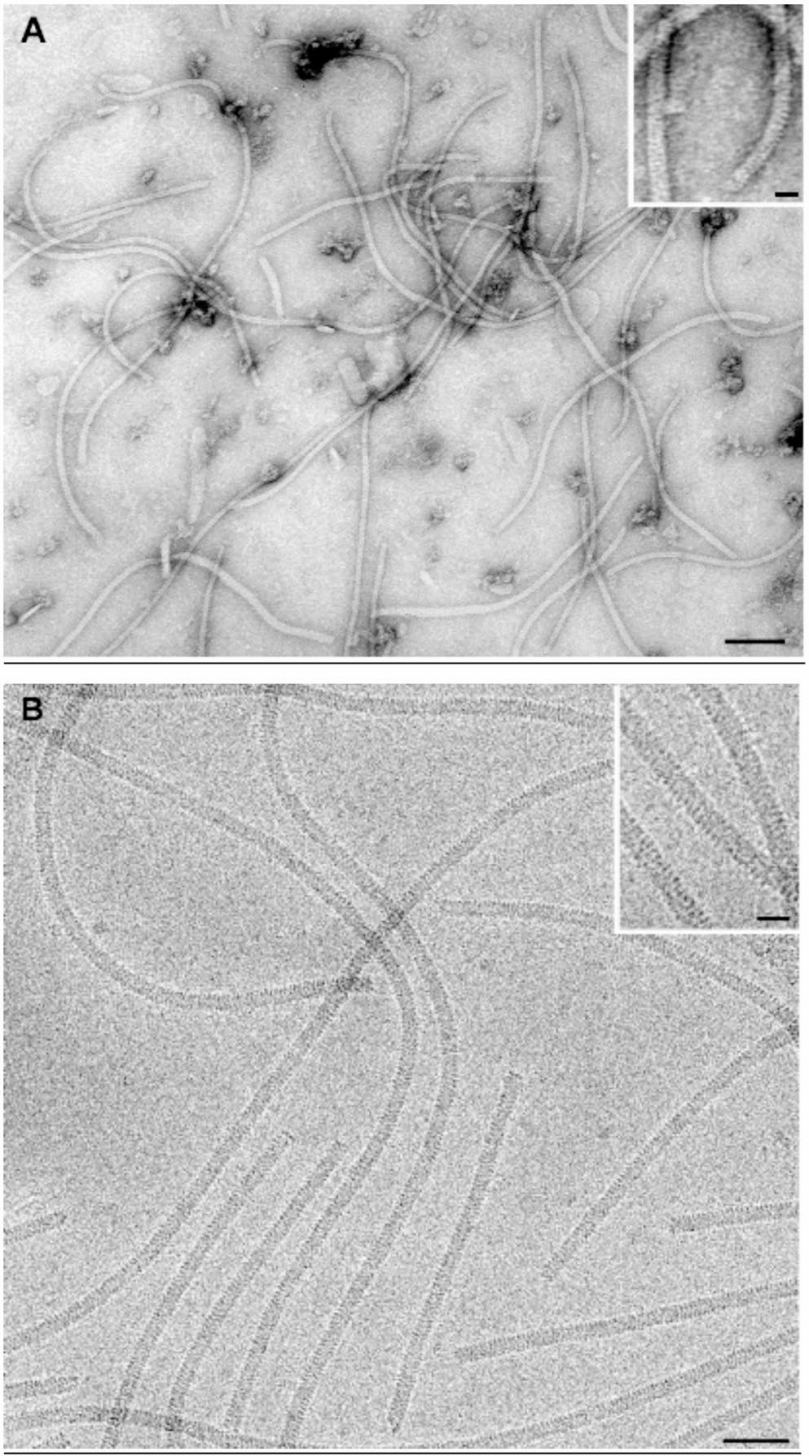
Structure of purified PPV virions observed using (**A**) transmission electron microscopy (TEM) and (**B**) cryo-EM. Insets show the fine structure of the helical capsid arrangement. bars: A, 100 nm, inset 10 nm; B, 50 nm, inset 25 nm

The presence of some residual protein and membrane aggregates was observed in the virus suspension. Virions were measured, and from a total of 50 particles, a modal length of 650 nm was determined (Fig. 1).

Sanger sequencing of an amplicon obtained from the 3’-terminal region of the PPV genome showed that the 990 nucleotides that were identified as the CP gene had 99% sequence identity to the corresponding sequences (top hits by BLASTn or BLASTp) of the PPV-M isolates PS (accession number AJ243957.1) and SK1 (accession number EF626549.1). The sequence from this study was deposited in the GenBank database (BankIt2983039 PPV-GR0046 PV955083; Supplementary Data 1).

### Filamentous PPV virions assemble into a helical heterooligomeric structure formed by CP and ssRNA

**Fig. 2:**
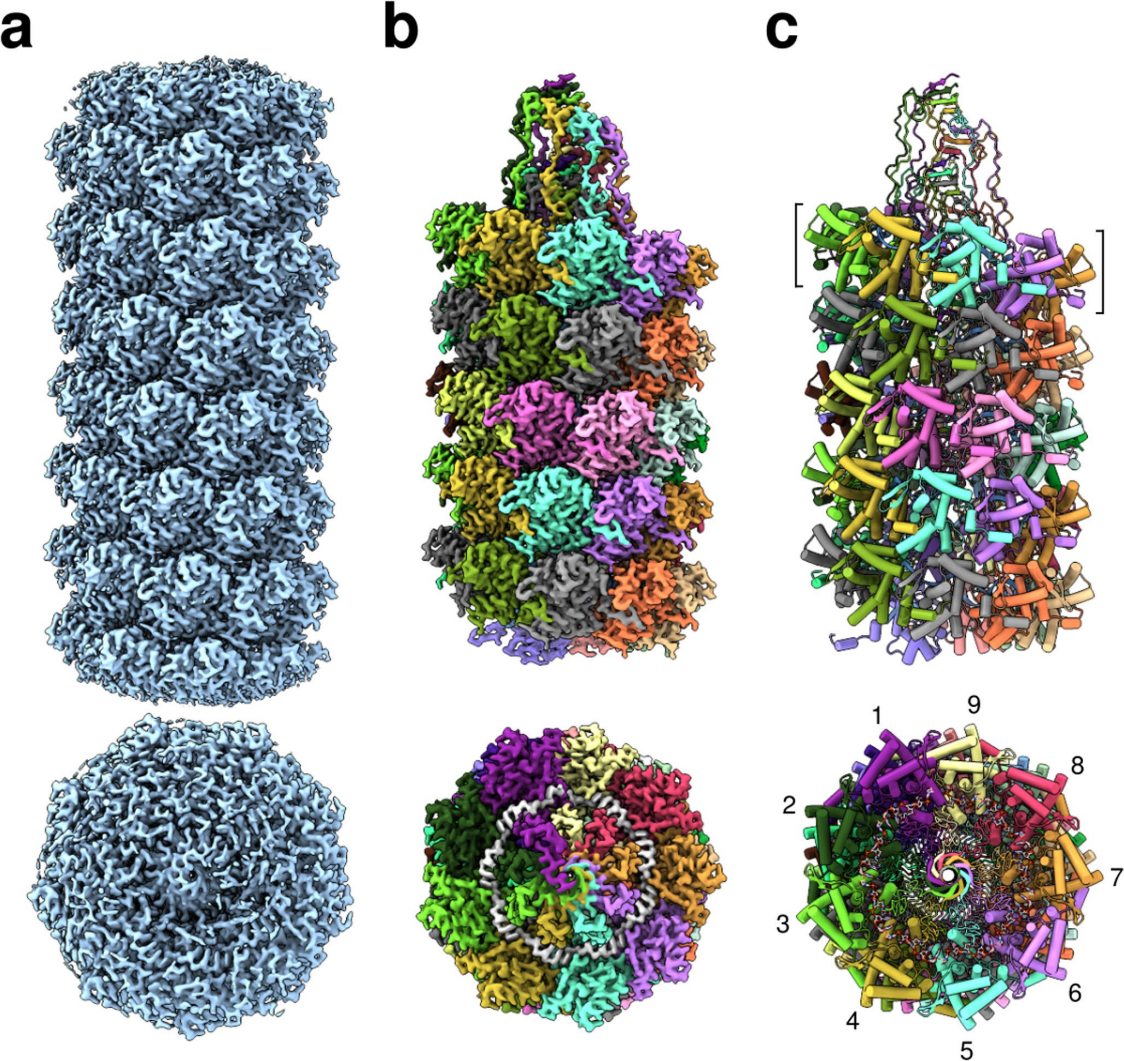
Structure of PPV virions. Maps and models in a side view and a top view are displayed at the top and bottom, respectively. **a** Reconstructed cryo-EM map. **b** Reconstructed cryo-EM map color coded according to the model, with the unmodeled regions omitted. The map corresponding to the ssRNA molecule is shown in white. **c** Cartoon representation of the model, with individual chains shown in different colors. The ssRNA is displayed as an atomic representation with separate colors for different heteroatoms. The bracket highlights one helical layer, and the numbers indicate the position of the CP protomer at each turn.

The structure of PPV virions was analyzed using cryo-EM, revealing filamentous objects with a diameter of 130 Å. The PPV structure was reconstructed at a resolution of 2.9 Å using a helical reconstruction approach (Fig. 2a), with a final helical symmetry characterized by a rise (ΔΖ) of 4.08 Å and a twist (ΔΦ) of −40.89° (Supplementary Fig. S1 and Supplementary Table S1). An initial model was generated in ModelAngelo [25], using the sequence of the coat protein (CP) and a polyuridine single-stranded RNA (ssRNA) sequence, and this model was subsequently refined using Coot and Phenix [26, 27] (Fig. 2b and c; Supplementary Table S1).

**Fig. 3:**
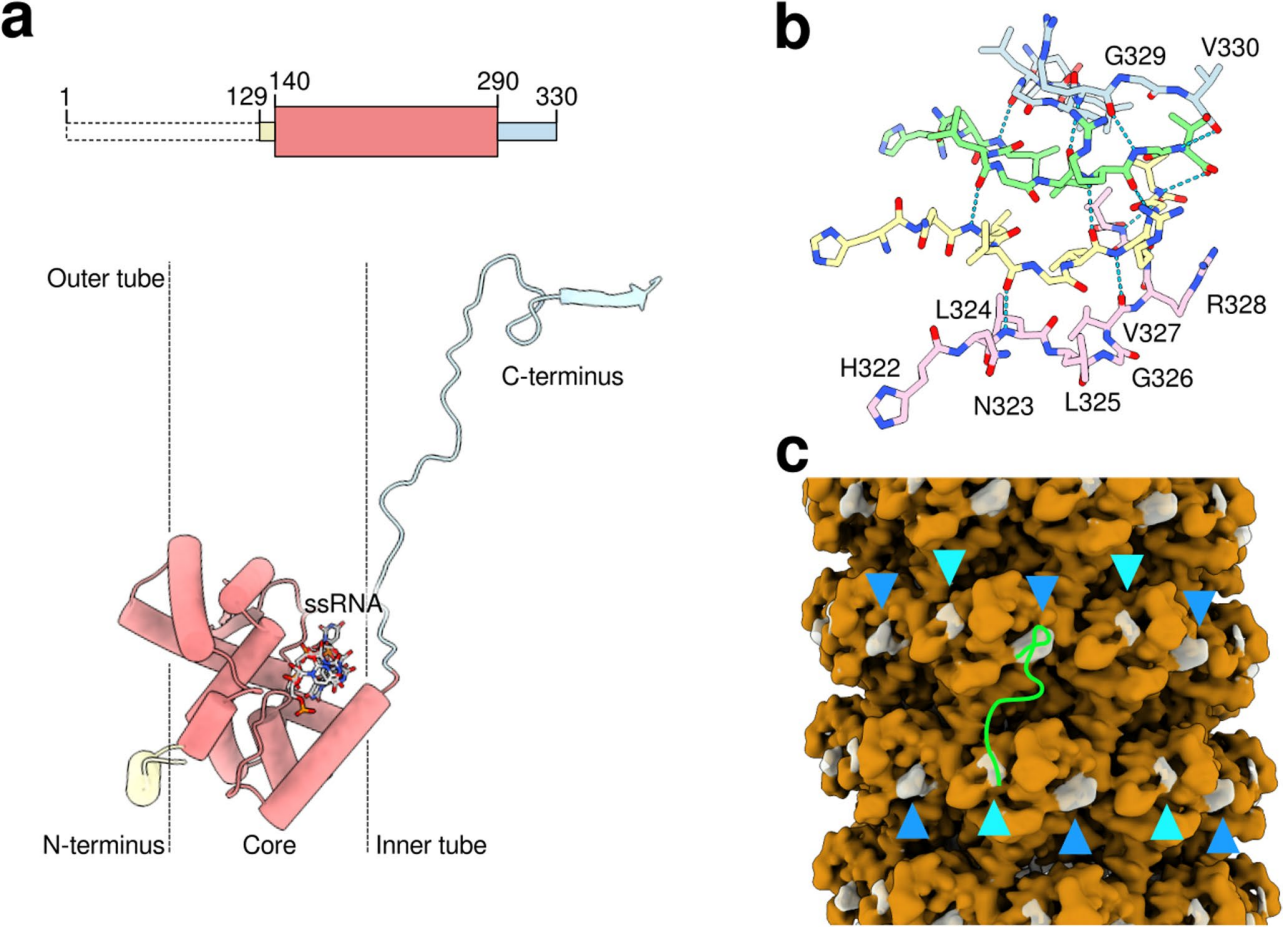
Structural analysis of PPV. **a** Detailed view of the asymmetric unit of the virion structure. The CP structure is represented as a ribbon diagram, while the ssRNA is shown as an atomic representation. The different domains of the CP are shown in different colors. The two dashed lines represent the hollow tube formed by the core of CP. A schematic representation of the primary sequence is included at the top. **b** The C-terminal beta-strands forming four layers. The hydrogen bonds between layers are represented as dashed lines, and amino acid residues are labeled. **c** cryo-EM map color-coded according to the structural model. The regions in brown represent the modeled part, while the grey semitransparent regions represent the unmodeled part. Symmetrically equivalent unmodeled regions are indicated by light blue or dark blue triangles. A green line representing the model of the N-terminus of the PVY is superimposed onto the map.

The PPV CP protein is organized in helical layers comprising 8.8 subunits per turn (Fig. 2c), encapsulating the ssRNA within the filament (Fig. 2b). The reconstruction was of sufficient quality to allow modeling of the residues Asn129 to Val330 (Fig. 3a). The CP adopts a fold consisting of a central globular domain (hereafter referred to as the core), encompassing residues Gln140 to Asn290, which is tightly packed to form a cylindrical, hollow tube. The intricate C-terminal region extends into and fills the inner part of the tube, engaging in extensive interactions with symmetry-related CP molecules. Notably, the last residues (Asn223 to Arg228) situated within the filament’s inner region partially adopt an amyloid fibril-like conformation, with only a single glycine residue deviating from this structural motif (Fig. 3b). Interestingly, the helical rise closely aligns with that observed in amyloid fibrils (3.75 Å). Conversely, a small portion of the N-terminal region (Asn129-Gln140) is positioned externally relative to the core tube and contributes to capsid stabilization through interactions with neighboring CP cores. The ssRNA is located within an armpit-shaped groove formed between the core and the C-terminal region of CP, accommodating five nucleotides per protomer (asymmetric unit). Notably, four nucleotides are oriented toward the base of the groove, while one (number 4) is oriented in the opposite direction.

The PPV structure exhibits a striking resemblance to that of potato virus Y (PVY) [23], which possesses a similar helical symmetry (ΔΖ: 3.95 Å and ΔΦ: −40.95°) and a root mean square deviation (RMSD) of 0.74 Å (between 166 residues considering Cα atoms). The primary structural difference lies in the N-terminal region, where the PVY model includes an extended segment comprising 22 additional residues. In the PPV reconstruction, two unmodeled density blobs closely correspond to two portions of the PVY N-terminus (Fig. 3c). This observation suggests that these residues may adopt a packing arrangement similar to that in PVY; however, they appear to be more flexible in our reconstructed sample.

### Special features of the plum pox virus coat protein

#### Post-translational modifications

Mass spectrometry analysis was done on the purified virions, starting with the same sample used for the cryo-EM analysis. A sample enriched for phosphate residues had 15 sites that were judged with high certainty to be phosphorylated, starting from residue 71 of the protein. In addition, three other phosphorylation sites were found with a slightly lower probability of modification.

A schematic representation of the coat protein sequence is shown in Supplementary Fig. 2, with the peptides containing modifications highlighted in green. One of the phosphorylated peptide fragments (amino acid residues 71–93) is also highly likely to contain a glycan of the type HexNAc [S/T].

In addition, mass spectrometry analysis showed that several large peptides corresponding to the N-terminal region appear to have at least one *O*-GlcNAc modification. Because some of the peptides generated by enzymatic digestion with trypsin were very large (Supplementary Fig. S3), we also carried out a mass spectrometric analysis in the acquisition range of 700–4000 as the m/z ratio, and the presence of high-molecular-weight peaks in this area (data not shown) is compatible with larger peptides, possibly bearing PTMs and covering the N-terminal region. Supplementary File 1 provides a list of peptides that were predicted with “high confidence” (nearly 100% probability) to contain modifications.

#### Amyloidogenic properties and a disordered portion

Amyloidogenic regions were predicted for PPV and five other potyvirus CPs, using AmylPred2 software with multiple algorithms, and the consensus produced six stretches of variable length, with the longest containing 14 amino acid residues (aa 171–184) (Supplementary Fig. S4). The alignment shows the correspondence of the predicted amyloidogenic regions for all six viruses (Supplementary Fig. S5). The amino acid sequence motif DAG, which is involved in aphid transmission, and the three-amino-acid motif described previously by Zamora et al. [24] to be a signature sequence of potyviruses, were identified in the alignment.

The *in silico* prediction of the intrinsically disordered portions of the CP provides a mapping of the segments that escaped the cryo-EM analysis. A total of five segments are predicted, two of which are longer than 30 amino acids. The portion of disordered residues in the protein is 29.39%. In total, 77 amino acids (23.3% of the total) within the first 100 amino acids of the N-terminal portion are disordered (Supplementary Fig. S6). This is in agreement with the lack of coverage by cryo-EM of the first 140 amino acids in the N-terminal portion of the CP. Moreover, intrinsically disordered or unstructured regions are apparent from the corresponding low-confidence prediction (pLDDT < 50) values determined for the AlphaFold-generated model (see the following paragraph).

#### AlphaFold-mediated simulation of folding

Folding simulations were performed using AlphaFold to verify the correspondence between the simulated model and the structure generated by cryo-EM analysis. Interestingly, the CP amino acid sequence, when used as a query against the AlphaFold Protein Structure Database, showed the highest structural homology with the CP of papaya ringspot virus, a potyvirus that infects *Carica papaya* (UniProt entry: B8Y3I5 _CARPA; [28]) (Supplementary Fig. S7). A complete list of alignment hits ranking in descending order of similarity is provided in Supplementary File 2. The AlphaFold Protein Structure Database features a limited number of structures related to potyvirus CPs. Nevertheless, we noticed that the second-highest-ranking hit shows similarity to the 3D structure of an RNA-directed RNA polymerase C-terminal-domain-containing protein (UniProt entry: Q5ZF65_PLAMJ) which has an RNA-binding motif and a “Poty_coat” domain [29].

The folding of the CP monomer with and without post-translational modifications and that of a series of CP multimers was simulated. In particular, the monomer containing all of the phosphorylated sites (Supplementary Fig. S8), thus representing the condition of the biological sample used for the cryo-EM analysis, produced the structure shown in Supplementary Fig. S9 (see also the structures deposited in the ModelArchive repository). The CP octamer yielded a structure closely resembling that determined by cryo-EM in the assembled virion, which is organized in helical layers with 8.8 subunits per turn (Supplementary Fig. S10). According to the output formatting rules of AlphaFold, low-order regions are shown, and the predicted structure typically has a ribbon-like appearance.

#### SDS-PAGE and Western blot analysis of the CP

The partially purified virus (roughly equivalent to the amount of virus in 3 g of infected plant tissues) was separated by SDS-PAGE (Supplementary Fig. S11A). Western blotting and detection using a polyclonal antibody showed a major denatured CP band (Supplementary Fig. S11B, lane 1). This band essentially migrated at the same rate (predicted MW, 36.5 kDa) as an unpurified virus CP from the soluble supernatant (Supplementary Fig. S11B, lane 2) or pellet (Supplementary Fig. S11B, lane 3) obtained from a small amount of infected tissue, while no reaction was observed when healthy tissue was tested (Supplementary Fig. S11B, lane 4). However, in the partially purified virus lane, several faster-migrating minor bands, which might have been degradation products of the CP, were also detected by the antibody.

## Discussion

This study provides a high-resolution structural view of plum pox virus virions using cryo-EM, contributing to the growing body of knowledge on the architecture of filamentous plant viruses within the family *Potyviridae*. Our findings reveal a helical structure assembled from CP subunits encapsidating a single-stranded RNA genome, consistent with the general model for potyviruses and related flexible filamentous viruses.

A striking observation across multiple structural studies of flexible filamentous plant viruses, including potyviruses such as watermelon mosaic virus (WMV) [24], potato virus Y (PVY) [23], turnip mosaic virus (TuMV) [20], and even viruses from other families such as potato virus X (PVX) of the family *Alphaflexiviridae* [21], is the remarkable conservation of the CP core fold. Despite low primary sequence similarity, the overall tertiary structure, rich in alpha-helices, is maintained. Our structure of PPV aligns with this observation, showing strong similarity to the published structures of WMV and PVY [20], suggesting a common evolutionary solution for assembling stable, yet flexible, virions. The helical parameters determined in our study are also comparable to those reported for other potyviruses such as WMV [24], PVY [23], and SPFMV [22], indicating conserved principles in subunit packing and overall filament geometry. Nevertheless, unlike that of PPV, the C-terminal region of the CPs of WMV and TuMV, which occupies the internal part of the virion, appears to be disordered.

The interaction between the CP and the viral ssRNA is of fundamental importance for virion integrity and assembly. Our structure visualizes the RNA molecule nested within a groove in the CP subunit, with each protomer accommodating approximately five nucleotides, a stoichiometry also observed in WMV [24] and PVX [21]. The conservation of key residues (Ser, Arg, and Asp in WMV; Arg and Ile in PVX) within this RNA-binding pocket across different viruses underscores its functional importance. The detailed conformation of the RNA within this groove, potentially showing base flipping or stacking, as seen in PVX [21], highlights the specific structural constraints imposed by the CP during encapsidation. Furthermore, studies on TuMV have demonstrated that CP-RNA interactions are crucial for the correct positioning of the CP N-terminal arm, which acts as a molecular staple, locking subunits together [20]. The requirement for RNA for stable particle formation is further supported by studies on PVX, which showed that assembly is dependent on RNA [21], and PPV, which showed that stable CP accumulation and particle formation appear to be linked to RNA replication competence [10]. The assembly is likely to initiate at specific origin of assembly sequences (OASs), possibly near the 5' end of the genome, as suggested for PPV and PVY [10, 23].

Consistent with findings for WMV [24] and PVY [23], the extreme N-terminal region of the PPV CP was not fully resolved in our cryo-EM map, suggesting inherent flexibility or disorder in this domain. This also applies to other potyviruses whose structures were published earlier.

The N-terminal portion of the CP is involved in the proper building and flexibility of the virion structure. In addition, mass spectrometry in the 700–4000 m/z range after trypsin digestion showed high-molecular-weight peaks compatible with possible O-GlcNAc modifications. However, for these large peptides, structural assignment remains uncertain due to potential additional PTMs. Furthermore, we cannot exclude the possibility that modifications in the N-terminal region might have gone undetected in our analysis, due to a different technical approach than those applied in previous investigations or to degradation of the externally exposed CP N-terminus. Indeed, our Western blot analysis of the partially purified CP suggested that some degradation had occurred during purification and storage of the virions.

Notably, the N-terminal conserved DAG motif (aa 11, 12, and 13), which interacts with a PTK motif at the C-terminus of HC-Pro to produce the transmission-competent bridge that is needed for aphid transmission [6], is contained in this flexible, non-structured, and most likely externally exposed and hydrophilic portion of the protein.

A relatively rich body of literature is available regarding the prediction of disordered protein structures and how they affect the stability and functions of proteins [30]. For example, Walter et al. [31], in a study to explore the mutational robustness of the potyviral protein VPg and its interactor eukaryotic initiation factor (eIF4E), compared the effects of random mutations on these proteins. VPg exhibits higher mutational robustness when compared to eIF4E, indicating that intrinsically disordered regions (IDRs) in VPg are more tolerant of mutations. IDRs in VPg play a crucial role in the ability of the virus to adapt to its host plant, and mutations in eIF4E have a more significant effect on its interaction with VPg, suggesting that ordered regions are less adaptable. Thus, the higher mutational robustness of the IDRs of VPg is likely to facilitate adaptive changes in the virus, thereby playing an important role in viral evolution. While this region is crucial for inter-subunit interactions [32], its flexibility might also be important for other functions of CP during the viral life cycle. Conversely, the C-terminal region of the CP of WMV contributes significantly to the inner part of the virion structure and inter-subunit contacts [24].

Beyond the core structure, the function and stability of potyvirus CPs are modulated by extensive PTMs. Studies on PPV have identified *O*-GlcNAcylation sites, primarily within the N-terminal region, which enhance viral infectivity and potentially affect virion structure by increasing protease resistance [13]. Notably, the PPV CP was the first protein of a plant virus and of a virus with a positive-strand RNA genome for which typical *O*-GlcNAcylation was unambiguously demonstrated. This modification occurs at serine (Ser) and/or threonine (Thr) residues, primarily in the N-terminal region of the protein [33]. Specific sites that have been identified include Thr19, Thr24, Thr41, Thr50, Thr53, Thr54, Thr58, and Ser65. The O-GlcNAc transferase (OGT) responsible for this modification in plants is named "SECRET AGENT" (SEC) [34]. Studies with SEC mutants show that the viral titer and the rate of viral spread are reduced when O-GlcNAcylation is impaired [13, 34]. Although O-GlcNAcylation is not always essential for virus viability, it significantly enhances viral infection [35], playing different roles in different host species. For instance, while a PPV mutant (CP7T/A) lacking O-GlcNAcylation sites in its CP was able to replicate in infected *Nicotiana clevelandii*, *Nicotiana benthamiana*, and *Prunus persica* plants without noticeable defects, it showed clear defects in *Arabidopsis thaliana*. In mixed infections in *A. thaliana*, the CP7T/A mutant was outcompeted by the wild-type virus, indicating a major role of O-GlcNAcylation in this host [13].

Phosphorylation has also been demonstrated on the PPV CP, coexisting with *O*-GlcNAcylation and showing crosstalk between the two modifications [14, 36]. These PTMs occur in different PPV strains and host plants, on both assembled and unassembled CP, suggesting a fundamental role in regulating CP functions. The combined effects of O-GlcNAcylation and phosphorylation of the N-terminal segment of the PPV CP have been proposed to allow fine-tuning of protein stability, thereby providing the appropriate amount of CP required at each step of viral infection [8, 14, 35, 36]. Phosphorylation in this region has been linked to CP degradation by the ubiquitin proteasome system, with O-GlcNAcylation potentially protecting against this degradation [8, 14]. While our structural analysis focused on the main polypeptide chain, the presence and location of these PTMs, particularly within the flexible N-terminal region, are likely to contribute to the fine-tuning of CP stability, assembly, and its other roles during infection [14, 19, 23]. For instance, phosphorylation of the PVA CP has been linked to regulation of the switch between translation and replication [12, 37].

It is noteworthy that the relevance of phosphorylation within conserved motifs (such as the casein kinase II, CK2, motif) can differ among potyvirus strains. For example, phosphorylation-related mutations affecting T306 of PPV-SwCM CP (strain C) cause a significant loss of fitness, whereas similar mutations at T304 of PPV-R CP (strain D) do not have a noticeable deleterious effect [8, 38]. The structural plasticity and complexity suggested by the overall PTM landscape and the unresolved N-terminus may underpin the ability of the CP to participate in diverse processes [38–40].

Interestingly, comparative studies have revealed differences in stability even between related viruses such as the potyvirus SPFMV and the ipomovirus SPMMV, which infect the same host [22]. Although their overall CP structures and helical organizations are similar, SPMMV particles exhibit higher thermal stability. This suggests that subtle structural differences, possibly involving inter-subunit contacts and potentially influenced by differences in PTM patterns, can have measurable effects on virion properties. An atomic model for the potexvirus bamboo mosaic virus (BaMV) has revealed that flexible N- and C-terminal extensions allow deformation while still maintaining structural integrity [19].

Among the identified PTMs, several occur within the resolved cryo-EM structure. Notably, Ser137 is positioned near two basic residues of an adjacent CP subunit, Lys236 and Arg237 (Supplementary Fig. S12a). Phosphorylation at this site could enhance inter-subunit packing through favorable electrostatic interactions, potentially contributing to increased virion stability. In contrast, Ser151 lies in proximity to two acidic residues from neighboring CPs, Glu205 and Glu240 (Supplementary Fig. S12b); phosphorylation here may introduce electrostatic repulsion, thereby destabilizing the virion. Thr254 is located on the inner surface of the CP tube and is not solvent-exposed, suggesting that partial disassembly of the virion might be required for phosphorylation to occur. This residue interacts with two oxygen atoms from the main polypeptide chain at the C-terminus (Supplementary Fig. S12c), and its phosphorylation is likely to disrupt this interaction. Similarly, Ser270 engages with the main-chain nitrogen atom of Met244 from an adjacent CP and the side chain of Gln140 within the same CP (Supplementary Fig. S12d); phosphorylation at this site is also expected to weaken inter-subunit contacts. Finally, Thr309, buried within the C-terminal region of the inner tube, interacts with Glu278 of a neighboring CP (Supplementary Fig. S12e). As with Thr254, phosphorylation at this site probably requires partial disassembly and may contribute to virion destabilization by disrupting stabilizing contacts.

Overall, these observations suggest that site-specific phosphorylation can differentially modulate virion stability, either by reinforcing inter-subunit interactions or by causing destabilization, depending on the local electrostatic environment and structural context.

In the present study, we provide evidence of the presence of amyloidogenic regions in the coat protein of PPV. Amyloid stretches, or prion-like domains (PrLDs), have been predicted in proteins from diverse organisms [41, 42], including microorganisms, plants [43–46], and viruses [47], but little is known about their role in plant viruses. One notable example is the apparent homology between the potato virus Y (PVY) nuclear inclusion b protein and the human amyloid-beta (Aβ) peptide [48]. These structures are hypothesized to be involved in various aspects of the viral life cycle, potentially contributing to pathogenesis through aggregation and cellular disruption and perhaps playing functional roles in virion assembly, stability, or interaction with the host environment as "functional amyloids" [49]. Viral amyloids have been discussed as potential targets of antiviral agents [50]. Experimental investigations are ongoing to assess the biological relevance of these computationally predicted regions in the context of plant virus infections.

Viral proteins are highly divergent, making sequence-based searches below 30% amino acid sequence identity difficult. Horizontal gene transfers create structural similarities that are relevant for predicting protein functions if detected. However, viral proteins have limited representation among experimentally determined structures in the Protein Data Bank (PDB) and are absent from the AlphaFold Protein Structure Database. This limitation hinders the structure-based understanding of their functions and explains why the AlphaFold-based search of structurally homologous coat proteins of the PPV CP returned few structures from other plant viruses [51]. In the present study, in addition to using AlphaFold to predict the structure of the PPV coat protein, we exploited an additional feature of the model that allows regions to be identified that are unstructured or intrinsically disordered, as these regions are expected to produce a low-confidence prediction (pLDDT < 50), and the predicted structure will have a ribbon-like appearance.

In conclusion, our high-resolution cryo-EM structure of PPV reinforces the concept of a conserved architectural blueprint for flexible filamentous plant viruses, particularly members of the family *Potyviridae*. It highlights the critical interplay between CP structure, CP-RNA interactions, and CP-CP contacts, mediated partly by the N-terminal arm, in maintaining virion integrity, and assisting in virus movement [52]. Understanding these structural details, combined with knowledge of PTMs and inherent protein flexibility, provides a more complete picture of how these viruses could assemble and function. This detailed structural information offers a valuable platform for future functional studies and for the rational design of antiviral strategies targeting virion assembly or stability that might be applicable to other members of the family *Potyviridae* and related viral families [21, 23].

## Supplementary Material

Below is the link to the electronic supplementary material

Supplementary Material 1 (PDF 186 KB)

Supplementary Material 2 (PDF 467 KB)

Supplementary Material 3 (PDF 196 KB)

Supplementary Material 4 (PDF 284 KB)

Supplementary Material 5 (PDF 1.01 MB)

Supplementary Material 6 (PDF 246 KB)

Supplementary Material 7 (PDF 568 KB)

Supplementary Material 8 (XLSX 30.1 KB)

Supplementary Material 9 (CSV 137 KB)

Supplementary Material 10 (PDF 194 KB)

Supplementary Material 11 (PDF 175 KB)

Supplementary Material 12 (PDF 329 KB)

Supplementary Material 13 (PDF 276 KB)

Supplementary Material 14 (PDF 255 KB)

Supplementary Material 15 (PDF 199 KB)

Supplementary Material 16 (PDF 322 KB)

Supplementary Material 17 (PDF 229 KB)

## Data Availability

Publicly available cryo-EM data used in this study are available in the wwPDB repository (https://www.wwpdb.org) with the PDB DOI 10.2210/pdb9QY3/pdb. The AlphaFold-generated models are available in ModelArchive (www.modelarchive.org) [71] under the accession codes 10.5452/ma-6ofeo, 10.5452/ma-nn1or, 10.5452/ma-2ko83, and 10.5452/ma-bapcg.
