# Supplementary material for: Structural characterization of plum pox virus by cryo-electron microscopy
Source: Arch Virol. 2025 Dec 1;171(1):11. doi: 10.1007/s00705-025-06473-5 (PMC12669337; doi:10.1007/s00705-025-06473-5)
Supplement: Supplementary file 1 — Supplementary Material 1 (PDF 186 KB) [file 705_2025_6473_MOESM1_ESM.pdf]

Structural characterization of plum pox virus (PPV) by cryo-EM

Archives of Virology

Diane Marie Valérie Jeanne Bonnet, Antonio Chaves-Sanjuan, Nicoletta Contaldo, Angelo De Stradis, Rosanna Caliandro, Angelantonio Minafra, Filippo Geuna*

***Corresponding author: filippo.geuna@unimi.it**
Department of Agricultural and Environmental Sciences (DISAA) - Università degli Studi di Milano, Milan, Italy

Supplementary Data 1. Nucleotide and protein sequence of the coat protein (CP) portion of PPV (isolate GR0046). The 990 bp corresponding to the 3'-terminal coding region (CP) are shown along with the amino acid sequence.

```
>PV955083.1 Plum pox virus isolate GR0046 coat protein gene, partial cds
GCTGATGAAAGGGAGGATGATGAAGAAGTTGATGCAGGAGGACCTACTGTGGTAACTGCACCGGCAGCAA
CTGTGGCAACGACTCAACCAGCTCCAGTGATACAACCTGCACCCCAAACCACAGCACCAATGTTCAACCC
CATTTTCACTCCAGCAACAACTCAGCCTGCGGTAAGACCAGTACCTCCAATTTCAGGGACCAAACCGCGG
TCTTTTGGAGTTTATGGAAATGAAGACGCATCACCTAGCACCTCAAACACTTTGGTGAATACAGGAAGGG
ATAGGGACGTCGATGCAGGATCGATTGGAACTTTCACAGTGCCACGCCTAAAAACAATGACATCGAAGTT
ATCTCTACCGAAGGTGAAGGGAAAGGCAATTATGAACTTAAATCATTTGGCACATTACAGTCCTGCACAA
GTTGACTTGTCAAACACACGAGCTCCACAATCCTGCTTCCAGACTTGGTATGAAGGAGTTAAGCGTGATT
ATGACGTCACAGATGAGGAAATGAGCATCATTTTGAATGGCTTGATGGTTTGGTGCATCGAAAACGGAAC
GTCTCCGAACATCAATGGAATGTGGGTGATGATGGATGGGGAGACACAAGTGGAGTATCCAATAAAGCCA
TTGTTGGATCACGCGAAACCCACTTTTAGACAAATTATGGCACATTTCAGTAACGTGGCTGAAGCGTATA
TTGAAAAACGAAATTACGAGAAAGCATACATGCCAAGGTATGGAATTCAGCGCAACCTGACAGATTACAG
TCTCGCCAGATACGCCTTTGATTTTTACGAAATGACTTCAACAACGCCTGTGCGTGCACGTGAAGCTCAT
ATACAGATGAAGGCAGCAGCATTGAGAAATGTTCAAAATCGTTTATTTGGCTTGGATGGAAACGTCGGAA
CACAAGAAGAGGACACAGAGAGGCACACCGCTGGTGACGTGAATCGCAACATGCACAACCTCCTCGGTGT
GAGGGGAGTG

>XXS07728.1 coat protein, partial [Plum pox virus]
ADEREDDEEVDAGGPTVVTAPAATVATTQPAPVIQPAPQTTAPMFNPIFTPATTQPAVRPVPPISGTKPR
SFGVYGNEDASPSTSNTLVNTGRDRDVDAGSIGTFTVPRLKTMTSKLSLPKVKGKAIMNLNHLAHYSPAQ
VDLSNTRAPQSCFQTWYEGVKRDYDVTDEEMSIILNGLMVWCIENGTSPNINGMWVMMDGETQVEYPIKP
LLDHAKPTFRQIMAHFSNVAEAYIEKRNYEKAYMPRYGIQRNLTDYSLARYAFDFYEMTSTTPVRAREAH
IQMKAAALRNVQNRLFGLDGNVGTQEEDTERHTAGDVNRNMHNLLGVRGV
```
