# Supplementary material for: Structural characterization of plum pox virus by cryo-electron microscopy
Source: Arch Virol. 2025 Dec 1;171(1):11. doi: 10.1007/s00705-025-06473-5 (PMC12669337; doi:10.1007/s00705-025-06473-5)
Supplement: Supplementary file 3 — Supplementary Material 3 (PDF 196 KB) [file 705_2025_6473_MOESM3_ESM.pdf]

Structural characterization of plum pox virus (PPV) by cryo-EM
Archives of Virology
Diane Marie Valérie Jeanne Bonnet, Antonio Chaves-Sanjuan, Nicoletta Contaldo, Angelo De Stradis, Rosanna Caliandro, Angelantonio Minafra, Filippo Geuna*

***Corresponding author: filippo.geuna@unimi.it**
Department of Agricultural and Environmental Sciences (DISAA) - Università degli Studi di Milano, Milan, Italy

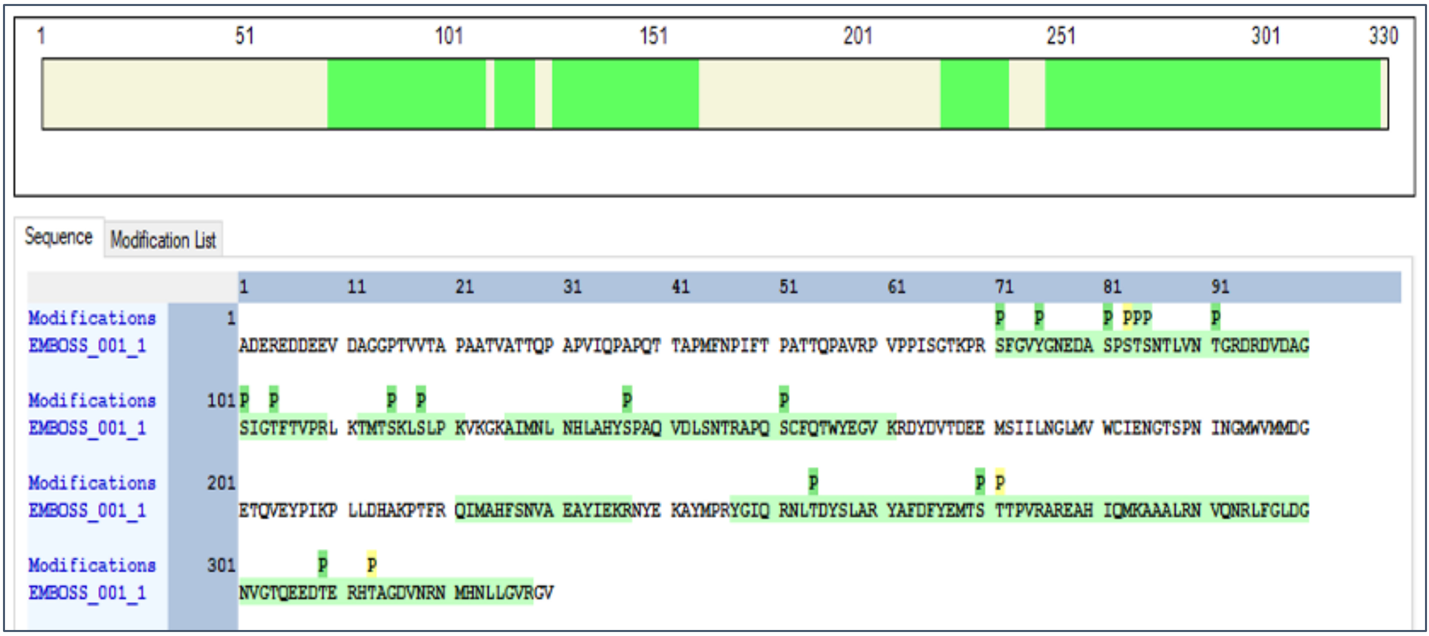

Supplementary Figure 2. Scheme of the coat protein aminoacid sequence with the post-translational modifications shown. P: phosphorylations; Green color shows the peptides identified by mass-spectrometry.
