# Supplementary material for: Structural characterization of plum pox virus by cryo-electron microscopy
Source: Arch Virol. 2025 Dec 1;171(1):11. doi: 10.1007/s00705-025-06473-5 (PMC12669337; doi:10.1007/s00705-025-06473-5)
Supplement: Supplementary file 4 — Supplementary Material 4 (PDF 284 KB) [file 705_2025_6473_MOESM4_ESM.pdf]

**Structural characterization of plum pox virus (PPV) by cryo-EM**
**Archives of Virology**
**Diane Marie Valérie Jeanne Bonnet, Antonio Chaves-Sanjuan, Nicoletta Contaldo, Angelo De Stradis, Rosanna Caliandro, Angelantonio Minafra, Filippo Geuna***

***Corresponding author: filippo.geuna@unimi.it**
**Department of Agricultural and Environmental Sciences (DISAA) - Università degli Studi di Milano, Milan, Italy**

# A

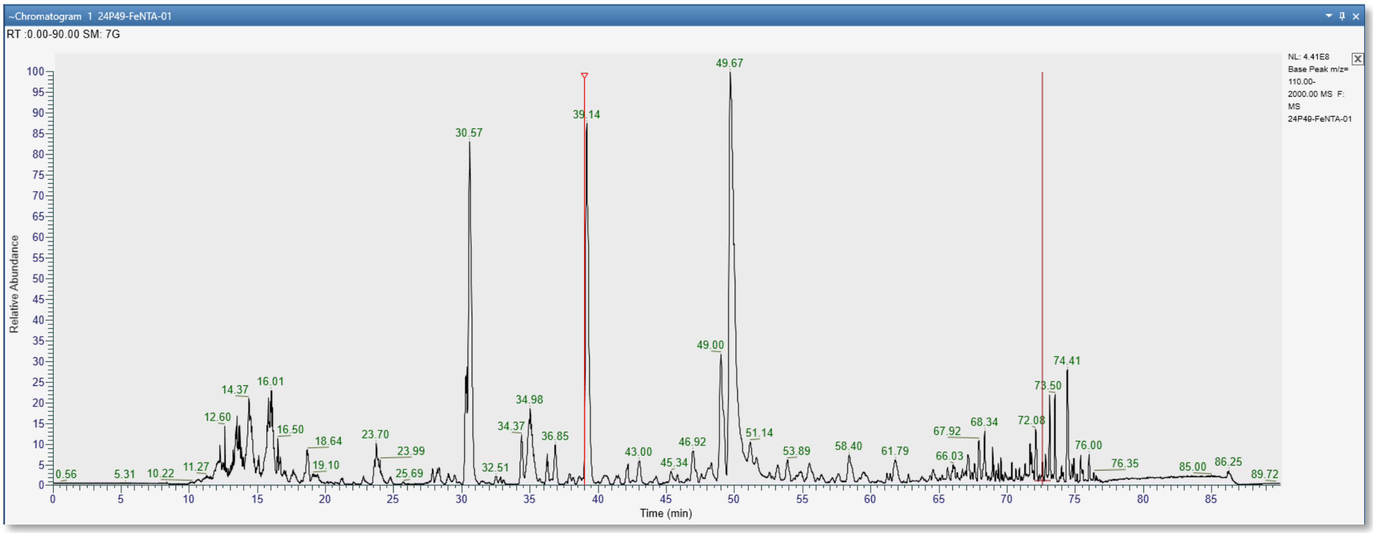


# B

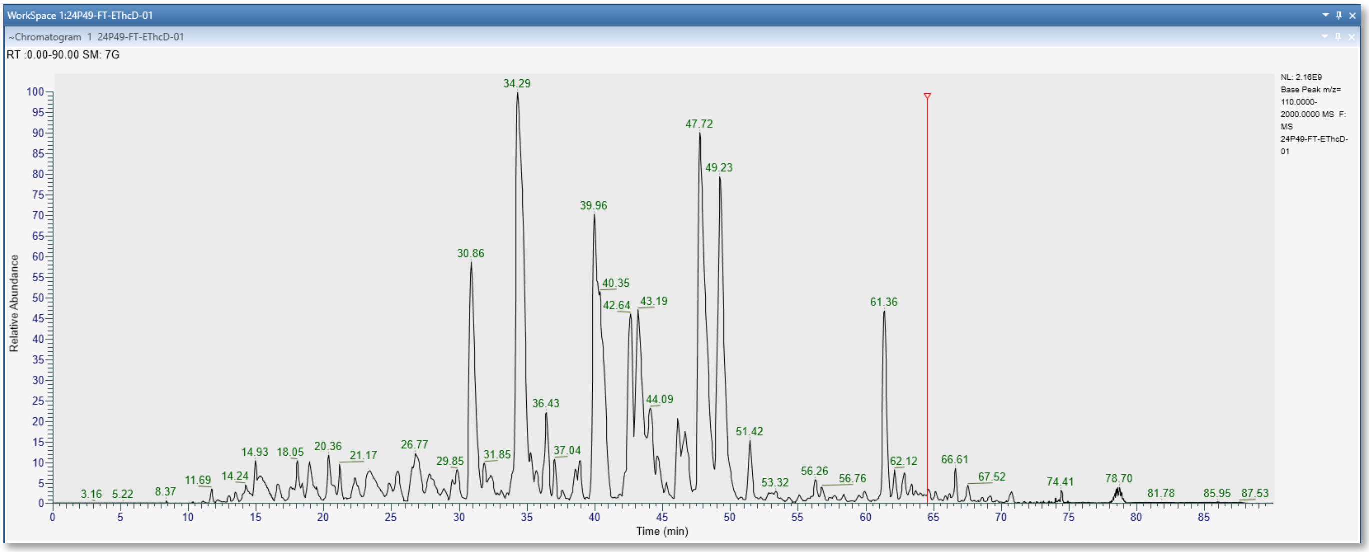


**Supplementary Figure 3.** nHPLC and mass spectrometry analysis. (A) chromatographic separation of tryptic digest enriched for phosphopeptides; (B) chromatographic separation of unenriched tryptic digest.
