# Supplementary material for: Structural characterization of plum pox virus by cryo-electron microscopy
Source: Arch Virol. 2025 Dec 1;171(1):11. doi: 10.1007/s00705-025-06473-5 (PMC12669337; doi:10.1007/s00705-025-06473-5)
Supplement: Supplementary file 5 — Supplementary Material 5 (PDF 1.01 MB) [file 705_2025_6473_MOESM5_ESM.pdf]

Documento pubblico redatto da Università degli Studi di Milano

Structural characterization of plum pox virus (PPV) by cryo-EM
Archives of Virology

Diane Marie Valérie Jeanne Bonnet, Antonio Chaves-Sanjuan, Nicoletta Contaldo, Angelo De Stradis, Rosanna Caliandro, Angelantonio Minafra, Filippo Geuna*

***Corresponding author: filippo.geuna@unimi.it**
Department of Agricultural and Environmental Sciences (DISAA) - Università degli Studi di Milano, Milan, Italy

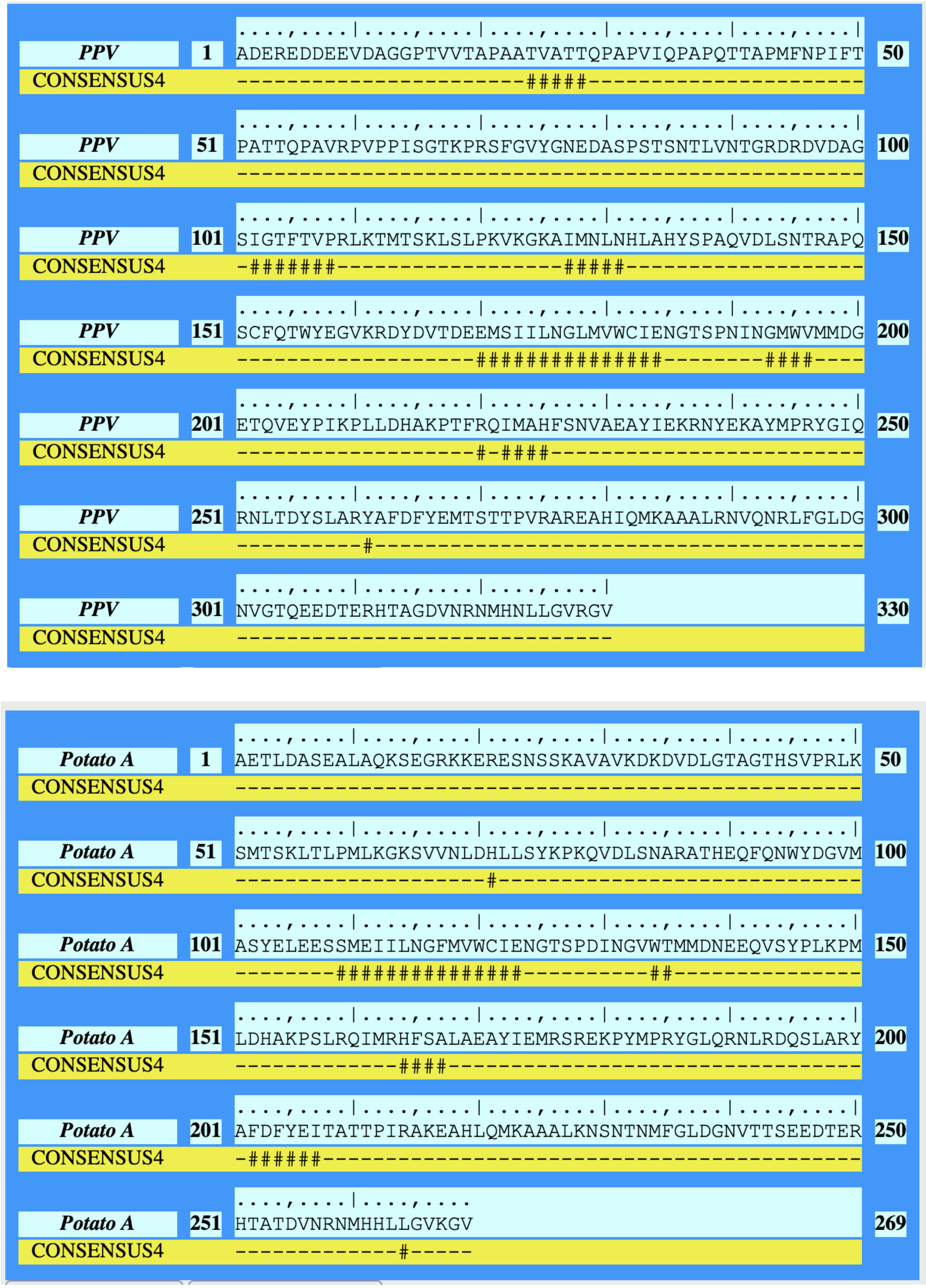

Supplementary Figure 4. *In silico* prediction of amyloidogenic regions of CP through the AmylPred2 software. **(A)** Output of the prediction.

Documento pubblico redatto da Università degli Studi di Milano

Structural characterization of plum pox virus (PPV) by cryo-EM

Archives of Virology

Diane Marie Valérie Jeanne Bonnet, Antonio Chaves-Sanjuan, Nicoletta Contaldo, Angelo De Stradis, Rosanna Caliandro, Angelantonio Minafra, Filippo Geuna*

***Corresponding author: filippo.geuna@unimi.it**

Department of Agricultural and Environmental Sciences (DISAA) - Università degli Studi di Milano, Milan, Italy

```
                    ....,....|....,....|....,....|....,....|....,....|
TuMV           1    AGETLDAGLTDEQKQAEKEKKEREKAEKERERQKQLALKKGKDVAQEEGK   50
CONSENSUS4          --------------------------------------------------

                    ....,....|....,....|....,....|....,....|....,....|
TuMV          51    RDKEVNAGTSGTFSVPRLKSLTSKMRVPRYEKRVALNLDHLILYTPEQTD  100
CONSENSUS4          -------------------------------------########-----

                    ....,....|....,....|....,....|....,....|....,....|
TuMV         101    LSNTRSTRKQFDTWFEGVMADYELTEDKMQIILNGLMVWCIENGTSPNIN  150
CONSENSUS4          ---------------------------###############--------

                    ....,....|....,....|....,....|....,....|....,....|
TuMV         151    GMWVMMDGDDQVEFPIKPLIDHAKPTFRQIMAHFSDVAEAYIEKRNQDRP  200
CONSENSUS4          ###------------------------#-##-------------------

                    ....,....|....,....|....,....|....,....|....,....|
TuMV         201    YMPRYGLQRNLTDMSLARYAFDFYEMTSRTPIRAREAHIQMKAAALRGAN  250
CONSENSUS4          --------------------------------------------------

                    ....,....|....,....|....,....|....,...
TuMV         251    NNLFGLDGNVGTTVENTERHTTEDVNRNMHNLLGVQGL              288
CONSENSUS4          --------------------------------------
```

```
                    ....,....|....,....|....,....|....,....|....,....|
SPFM           1    SSERTEFKDAGANPPAPKPQNIPPPPTITEVTDPEDPKQAALRAARAKQP   50
CONSENSUS4          --------------------------------------------------

                    ....,....|....,....|....,....|....,....|....,....|
SPFM          51    ATIPESYGRDTSKEKESIVGASSKGARDKDVNVGTVGTFVVPRVKMNANK  100
CONSENSUS4          --------------------------------##########--------

                    ....,....|....,....|....,....|....,....|....,....|
SPFM         101    KRQPMVNGRAIINFQHLSTYEPEQFEVANTRSTQEQFQAWYEGVKGDYGV  150
CONSENSUS4          ---------########---------------------------------

                    ....,....|....,....|....,....|....,....|....,....|
SPFM         151    DDTGMGILLNGLMVWCIENGTSPNINGVWTMMDGDEQVTYPIKPLLDHAV  200
CONSENSUS4          ----#############---------#####-------------------

                    ....,....|....,....|....,....|....,....|....,....|
SPFM         201    PTFRQIMTHFSDVAEAYIEMRNRTKAYMPRYGLQRNLTDMSLARYAFDFY  250
CONSENSUS4          --##-####-----------------------------------------

                    ....,....|....,....|....,....|....,....|....,....|
SPFM         251    ELHSTTPARAKEAHLQMKAAALKNAKNRLFGLDGNVSTQEEDTERHTTTD  300
CONSENSUS4          --------------------------------------------------

                    ....,....|....,
SPFM         301    VTRNIHNLLGMRGVQ                                     315
CONSENSUS4          -------##------
```

Supplementary Figure 4. *In silico* prediction of amyloidogenic regions of CP through the AmylPred2 software. **(A)** Output of the prediction. *(continued)*

Documento pubblico redatto da Università degli Studi di Milano

Structural characterization of plum pox virus (PPV) by cryo-EM

Archives of Virology

Diane Marie Valérie Jeanne Bonnet, Antonio Chaves-Sanjuan, Nicoletta Contaldo, Angelo De Stradis, Rosanna Caliandro, Angelantonio Minafra, Filippo Geuna*

***Corresponding author: filippo.geuna@unimi.it**
Department of Agricultural and Environmental Sciences (DISAA) - Università degli Studi di Milano, Milan, Italy

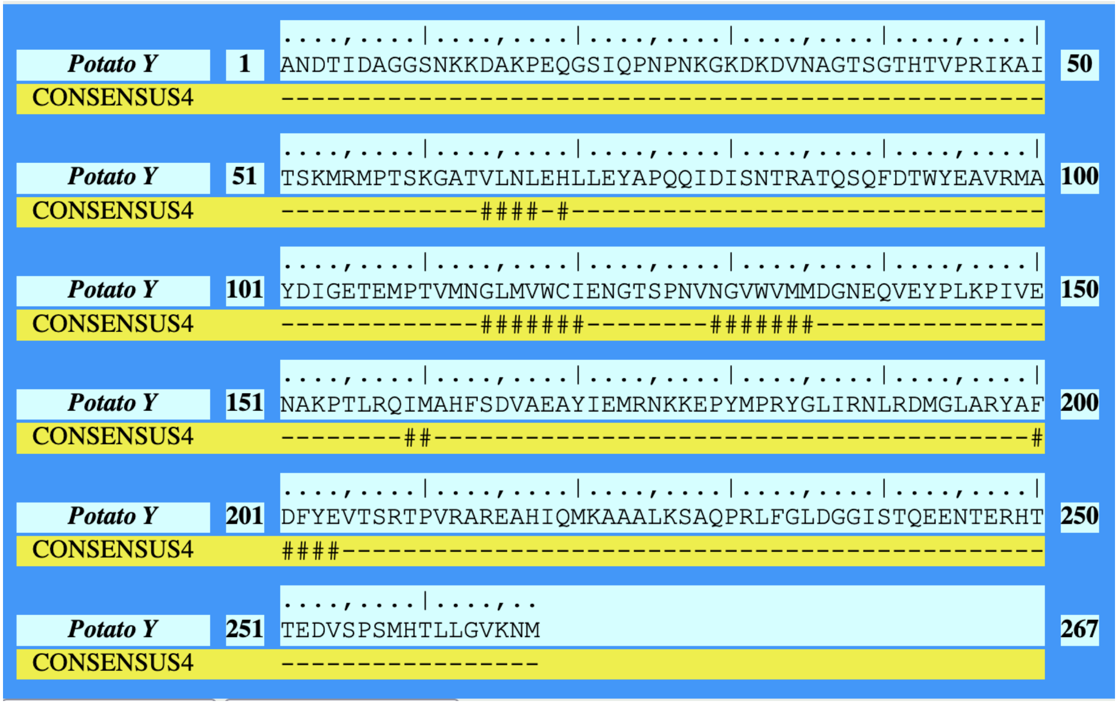

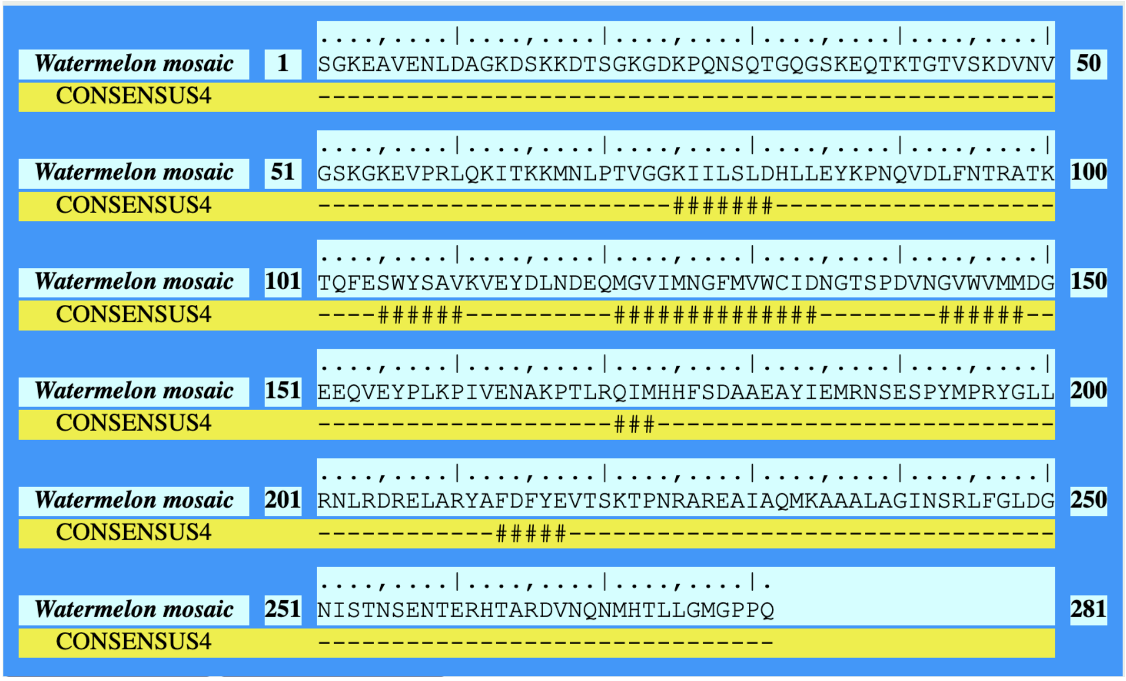

Supplementary Figure 4. *In silico* prediction of amyloidogenic regions of CP through the AmylPred2 software. **(A)** Output of the prediction. *(continued)*
