# Supplementary material for: Structural characterization of plum pox virus by cryo-electron microscopy
Source: Arch Virol. 2025 Dec 1;171(1):11. doi: 10.1007/s00705-025-06473-5 (PMC12669337; doi:10.1007/s00705-025-06473-5)
Supplement: Supplementary file 6 — Supplementary Material 6 (PDF 246 KB) [file 705_2025_6473_MOESM6_ESM.pdf]

Structural characterization of plum pox virus (PPV) by cryo-EM
Archives of Virology

Diane Marie Valérie Jeanne Bonnet, Antonio Chaves-Sanjuan, Nicoletta Contaldo, Angelo De Stradis, Rosanna Caliandro, Angelantonio Minafra, Filippo Geuna*

***Corresponding author: filippo.geuna@unimi.it**
Department of Agricultural and Environmental Sciences (DISAA) - Università degli Studi di Milano, Milan, Italy

```
SPFMV    SS--ERTEFKDAGAN-----------PPAPKPQNIPPPPTITE----VTDPEDPKQAALR    43
PVA      ------------------------------------------------------------    0
PPV      ADEREDDEEVDAGGPTVVTAPAATVATTQPAPVIQPAPQTTAPMFNPIFTPATT-QPAVR    59
TuMV     ------------------------------------------------------------    0
WMV      --------------------------------------------------------SGKE    4
PVY      ------------------------------------------------------------    0

SPFMV    AARAKQ--PATIPESYGRD---------------TSKEKESIVGASSKGARDKDVNVGTV    86
PVA      -AETLDASEALAQKSEGRK-----------------KERE-SNSSKAVAVKDKDVDLGTA    41
PPV      PVPPISGTKPRSFGVYGNE-----------------DASPSTSNTLVNTGRDRDVDAGSI    102
TuMV     AGETLDAGLTDEQKQAEKEKKEREKAEKERERQKQLALKKGKDVAQEEGKRDKEVNAGTS    60
WMV      AVENLDAGKDSKKDTSGKGD----------KPQNSQTGQGSKEQTKTGTVSKDVNVGSK    53
PVY      ANDTIDAGGSNKKD--------------------AKPEQGSIQPNPNKGKDKDVNAGTS    39
              .                                             .::*: *:

SPFMV    GTFVVPRVKMNANKKRQPMVNGRAIINFQHLSTYEPEQFEVANTRSTQEQFQAWYEGVKG    146
PVA      GTHSVPRLKSMTSKLTLPMLKGKSVVNLDHLLSYKPKQVDLSNARATHEQFQNWYDGVMA    101
PPV      GTFTVPRLKTMTSKLSLPKVKGKAIMNLNHLAHYSPAQVDLSNTRAPQSCFQTWYEGVKR    162
TuMV     GTFSVPRLKSLTSKMRVPRYEKRVALNLDHLILYTPEQTDLSNTRSTRKQFDTWFEGVMA    120
WMV      G-KEVPRLQKITKKMNLPTVGGKIILSLDHLLEYKPNQVDLFNTRATKTQFESWYSAVKV    112
PVY      GTHTVPRIKAITSKMRMPTSKGATVLNLEHLLEYAPQQIDISNTRATQSQFDTWYEAVRM    99
         *   ***::  :.*   *        :.::**  * * * :: *:*: :  *: *:..*

SPFMV    DYGVDDTGMGILLNGLMVWCIENGTSPNINGVWTMMDGDEQVTYPIKPLLDHAVPTFRQI    206
PVA      SYELEESSMEIILNGFMVWCIENGTSPDINGVWTMMDNEEQVSYPLKPMLDHAKPSLRQI    161
PPV      DYDVTDEEMSIILNGLMVWCIENGTSPNINGMWVMMDGETQVEYPIKPLLDHAKPTFRQI    222
TuMV     DYELTEDKMQIILNGLMVWCIENGTSPNINGMWVMMDGDDQVEFPIKPLIDHAKPTFRQI    180
WMV      EYDLNDEQMGVIMNGFMVWCIDNGTSPDVNGVWVMMDGEEQVEYPLKPIVENAKPTLRQI    172
PVY      AYDIGETEMPTVMNGLMVWCIENGTSPNVNGVWVMMDGNEQVEYPLKPIVENAKPTLRQI    159
          * : :  *  ::**:*****:*****::**:*.***.: ** :*:**::::* *::***

SPFMV    MTHFSDVAEAYIEMRNRTKAYMPRYGLQRNLTDMSLARYAFDFYELHSTTPARAKEAHLQ    266
PVA      MRHFSALAEAYIEMRSREKPYMPRYGLQRNLRDQSLARYAFDFYEITATTPIRAKEAHLQ    221
PPV      MAHFSNVAEAYIEKRNYEKAYMPRYGIQRNLTDYSLARYAFDFYEMTSTTPVRAREAHIQ    282
TuMV     MAHFSDVAEAYIEKRNQDRPYMPRYGLQRNLTDMSLARYAFDFYEMTSRTPIRAREAHIQ    240
WMV      MHHFSDAAEAYIEMRNSESPYMPRYGLLRNLRDRELARYAFDFYEVTSKTPNRAREAIAQ    232
PVY      MAHFSDVAEAYIEMRNKKEPYMPRYGLIRNLRDMGLARYAFDFYEVTSRTPVRAREAHIQ    219
         * ***  ****** *.    ******: *** *  *********: : ** **:**  *

SPFMV    MKAAALKNAKNRLFGLDGNVSTQEEDTERHTTTDVTRNIHNLLGMRGVQ  315
PVA      MKAAALKNSNTNMFGLDGNVTTSEEDTERHTATDVNRNMHHLLGVKGV-  269
PPV      MKAAALRNVQNRLFGLDGNVGTQEEDTERHTAGDVNRNMHNLLGVRGV-  330
TuMV     MKAAALRGANNNLFGLDGNVGTTVENTERHTTEDVNRNMHNLLGVQGL-  288
WMV      MKAAALAGINSRLFGLDGNISTNSENTERHTARDVNQNMHTLLGMGPPQ  281
PVY      MKAAALKSAQPRLFGLDGGISTQEENTERHTTEDVSPSMHTLLGVKNM-  267
         ****** . : .:*****.: *  *:*****: **. .:* ***:
```

Supplementary Figure 5. *In silico* prediction of amyloidogenic regions of CP through the AmylPred2 software. **(B)** CLUSTAL Omega at: ebi.ac.uk (1.2.4) multiple sequence alignment with predicted regions highlighted in colors. The conserved aminoacid residues of Zamora et al. (2017) are shown by blue blocks.
