# Supplementary material for: Structural characterization of plum pox virus by cryo-electron microscopy
Source: Arch Virol. 2025 Dec 1;171(1):11. doi: 10.1007/s00705-025-06473-5 (PMC12669337; doi:10.1007/s00705-025-06473-5)
Supplement: Supplementary file 7 — Supplementary Material 7 (PDF 568 KB) [file 705_2025_6473_MOESM7_ESM.pdf]

Structural characterization of plum pox virus (PPV) by cryo-EM
Archives of Virology

Diane Marie Valérie Jeanne Bonnet, Antonio Chaves-Sanjuan, Nicoletta Contaldo, Angelo De Stradis, Rosanna Caliandro, Angelantonio Minafra, Filippo Geuna*

***Corresponding author: filippo.geuna@unimi.it**
Department of Agricultural and Environmental Sciences (DISAA) - Università degli Studi di Milano, Milan, Italy

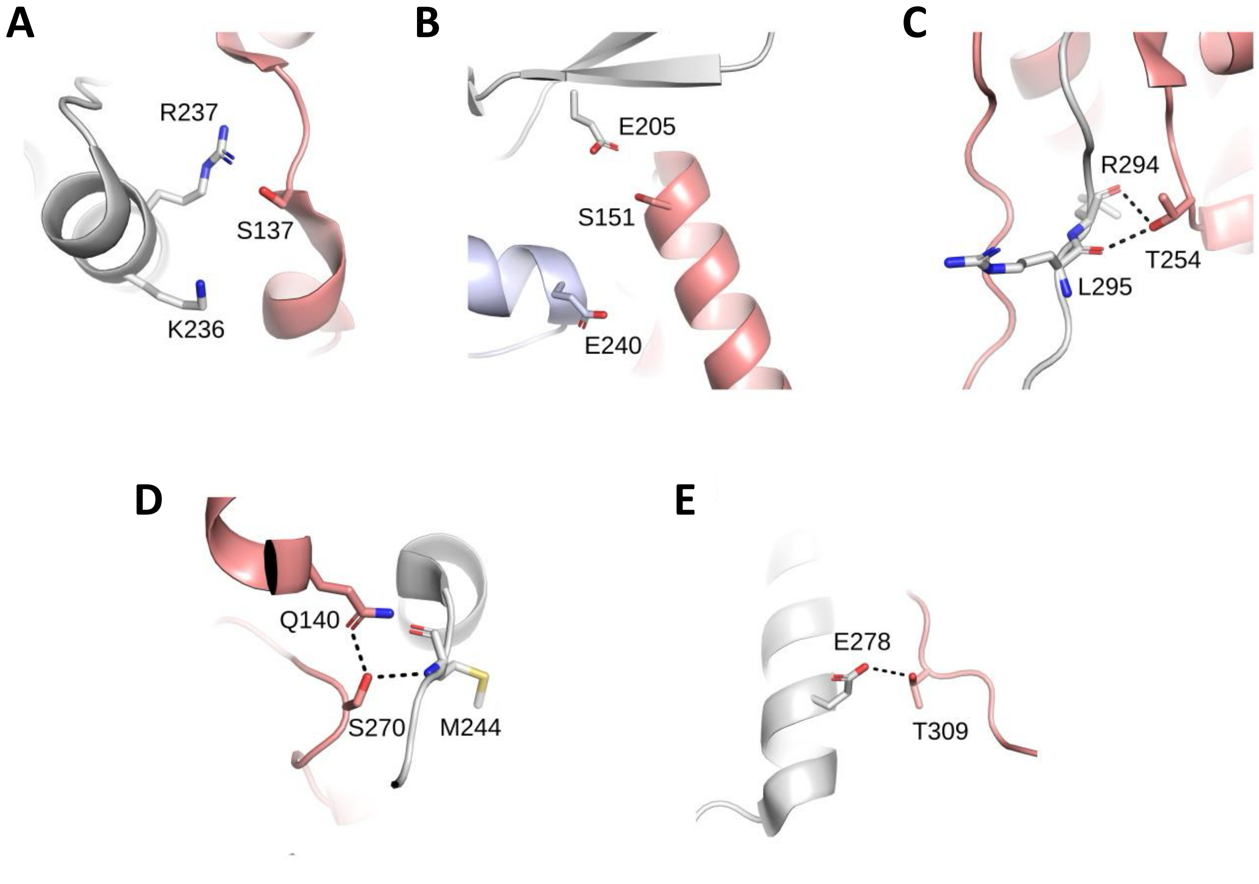

Supplementary Figure 12. Key interactions of the identified phosphorylable residues. The phosphorylable residues are on the protein colored in red, while neighbouring CP chains were coloured in white or purple. The residues were labeled and key interactions highlighted with dashes lines.
