# Supplementary material for: Structural characterization of plum pox virus by cryo-electron microscopy
Source: Arch Virol. 2025 Dec 1;171(1):11. doi: 10.1007/s00705-025-06473-5 (PMC12669337; doi:10.1007/s00705-025-06473-5)
Supplement: Supplementary file 10 — Supplementary Material 10 (PDF 194 KB) [file 705_2025_6473_MOESM10_ESM.pdf]

Documento pubblico redatto da Università degli Studi di Milano

Structural characterization of plum pox virus (PPV) by cryo-EM

Archives of Virology

Diane Marie Valérie Jeanne Bonnet, Antonio Chaves-Sanjuan, Nicoletta Contaldo, Angelo De Stradis, Rosanna Caliandro, Angelantonio Minafra, Filippo Geuna*

***Corresponding author: filippo.geuna@unimi.it**
Department of Agricultural and Environmental Sciences (DISAA) - Università degli Studi di Milano, Milan, Italy

Supplementary Table 1. Cryo-EM data collection, refinement and validation statistics.

**Data collection and processing**	
Structure name	PPV
Microscope	TALOS Arctica
Voltage (kV)	200
Camera	Falcon 3
Magnification	× 120,000
Total electron dose ($e^-/Å^2$)	40.0
Defocus range (μm)	-0.5 and -2.2 μm
Pixel size (Å)	0.889
Micrographs	2508
Manually picked filaments	29892
Initial segments	406769
Final segments	357073
Helical Symmetry	
Rise ΔZ (Å)	4.08
Twist ΔΦ (°)	-40.89
Resolution (Å)	2.89
(FSC threshold)	(0.143)
Sharpening B-factor ($Å^2$)	-139.7
EMDB code	EMD-53450
Model refinement	
CP molecules	45
Protein residues	9090
RNA nucleotides	225
r.m.s. deviations	
Bond lengths (Å)	0.006
Bond angles (°)	0.641
Ramachandran plot	
Favored (%)	95.02
Allowed (%)	4.98
Disallowed (%)	0.00
Validation	
Molprobity score	1.47
Clashscore	3.33
Poor rotamers (%)	0.00
Map-model correlation (CCmask)	0.92
PDB code	9QY3
