# Supplementary material for: Structural characterization of plum pox virus by cryo-electron microscopy
Source: Arch Virol. 2025 Dec 1;171(1):11. doi: 10.1007/s00705-025-06473-5 (PMC12669337; doi:10.1007/s00705-025-06473-5)
Supplement: Supplementary file 11 — Supplementary Material 11 (PDF 175 KB) [file 705_2025_6473_MOESM11_ESM.pdf]

Structural characterization of plum pox virus (PPV) by cryo-EM
Archives of Virology
Diane Marie Valérie Jeanne Bonnet, Antonio Chaves-Sanjuan, Nicoletta Contaldo, Angelo De Stradis, Rosanna Caliandro, Angelantonio Minafra, Filippo Geuna*

***Corresponding author: filippo.geuna@unimi.it**
Department of Agricultural and Environmental Sciences (DISAA) - Università degli Studi di Milano, Milan, Italy

Supplementary Table 2. nLC-MS/MS liquid chromatography elution profile.

N.	Time	Flow (µL/min)	%B
1	0.000	*Run*	
2	0.000	0.300	4.0
3	3.000	0.300	4.0
4	55.000	0.300	25.0
5	63.000	0.300	40.0
6	73.000	0.300	99.0
7	83.000	0.300	99.0
8	85.000	0.300	4.0
17	New Row		
18	90.000	*Stop run*	

N: elution step; %**B**: concentration of the B mobile-phase eluant (0.1% formic acid in acetonitrile, v/v)
