# Supplementary material for: Structural characterization of plum pox virus by cryo-electron microscopy
Source: Arch Virol. 2025 Dec 1;171(1):11. doi: 10.1007/s00705-025-06473-5 (PMC12669337; doi:10.1007/s00705-025-06473-5)
Supplement: Supplementary file 13 — Supplementary Material 13 (PDF 276 KB) [file 705_2025_6473_MOESM13_ESM.pdf]

Structural characterization of plum pox virus (PPV) by cryo-EM
Archives of Virology

Diane Marie Valérie Jeanne Bonnet, Antonio Chaves-Sanjuan, Nicoletta Contaldo, Angelo De Stradis, Rosanna Caliandro, Angelantonio Minafra, Filippo Geuna*

***Corresponding author: filippo.geuna@unimi.it**
Department of Agricultural and Environmental Sciences (DISAA) - Università degli Studi di Milano, Milan, Italy

```
Sequence match      HSP score: 929        E-value: 4.22206e-122

                    Identity: 67%         Positives: 208/256 (81%)     Gaps: 6/256 (2%)

Your query     1  GVYGNEDASPSTSNTLVNTG-RDRDVDAGSIGTFTVPRLKTMTSKLSLPKVKGKAIMNLN  60
                  G     D S ST      TG RDRDV+ G+ GTFTVPR+K+ T K+ LP++KGK ++NLN
B8Y3I5         1  GASDGNDVSTSTK-----TGERDRDVNVGTSGTFTVPRIKSFTDKMVLPRIKGKTVLNLN  60

Your query    61  HLAHYSPAQVDLSNTRAPQSCFQTWYEGVKRDYDVTDEEMSIILNGLMVWCIENGTSPNI  120
                  HL  Y+P Q+D+SNTRA  S F+ WYEGV+ DY + D EM ++LNGLMVWCIENGTSP+I
B8Y3I5        61  HLLQYNPQQIDISNTRATHSQFEKWYEGVRNDYGLNDNEMQVMLNGLMVWCIENGTSPDI  120

Your query   121  NGMWVMMDGETQVEYPIKPLLDHAKPTFRQIMAHFSNVAEAYIEKRNYEKAYMPRYGIQR  180
                  +G+WVMMDGETQV+YPIKPL++HA P+FRQIMAHFSN AEAYI KRN  + YMPRYGI+R
B8Y3I5       121  SGVWVMMDGETQVDYPIKPLIEHATPSFRQIMAHFSNAAEAYIAKRNATERYMPRYGIKR  180

Your query   181  NLTDYSLARYAFDFYEMTSTTPVRAREAHIQMKAAALRNVQNRLFGLDGNVGTQEEDTER  240
                  NLTD SLARYAFDFYE+ S TP RAREAH+QMKAAALRN   ++FG+DG+V  +EE+TER
B8Y3I5       181  NLTDISLARYAFDFYEVNSKTPDRAREAHMQMKAAALRNTSRKMFGMDGSVSNKEENTER  240

Your query   241  HTAGDVNRNMHNLLGVR  257
                  HT  DVNR+MH+LLG+R
B8Y3I5       241  HTVEDVNRDMHSLLGMR  257
```

Source: https://alphafold.ebi.ac.uk/entry/B8Y3I5

Supplementary Figure 7. PPV coat protein (CP) structural homology. Amino acid sequence alignment with the highest-ranking protein structure in the AlphaFold Protein Structure Database (UniProt entry: B8Y3I5 _CARPA) (AFDB, accessed March 13th 2025). This protein represents the papaya ringspot virus CP transgenically-inserted in papaya plants and resequenced (see ref. [28], Suzuki et al. 2008)
