# Supplementary material for: Structural characterization of plum pox virus by cryo-electron microscopy
Source: Arch Virol. 2025 Dec 1;171(1):11. doi: 10.1007/s00705-025-06473-5 (PMC12669337; doi:10.1007/s00705-025-06473-5)
Supplement: Supplementary file 15 — Supplementary Material 15 (PDF 199 KB) [file 705_2025_6473_MOESM15_ESM.pdf]

Structural characterization of plum pox virus (PPV) by cryo-EM

Archives of Virology

Diane Marie Valérie Jeanne Bonnet, Antonio Chaves-Sanjuan, Nicoletta Contaldo, Angelo De Stradis, Rosanna Caliandro, Angelantonio Minafra, Filippo Geuna*

***Corresponding author: filippo.geuna@unimi.it**

Department of Agricultural and Environmental Sciences (DISAA) - Università degli Studi di Milano, Milan, Italy

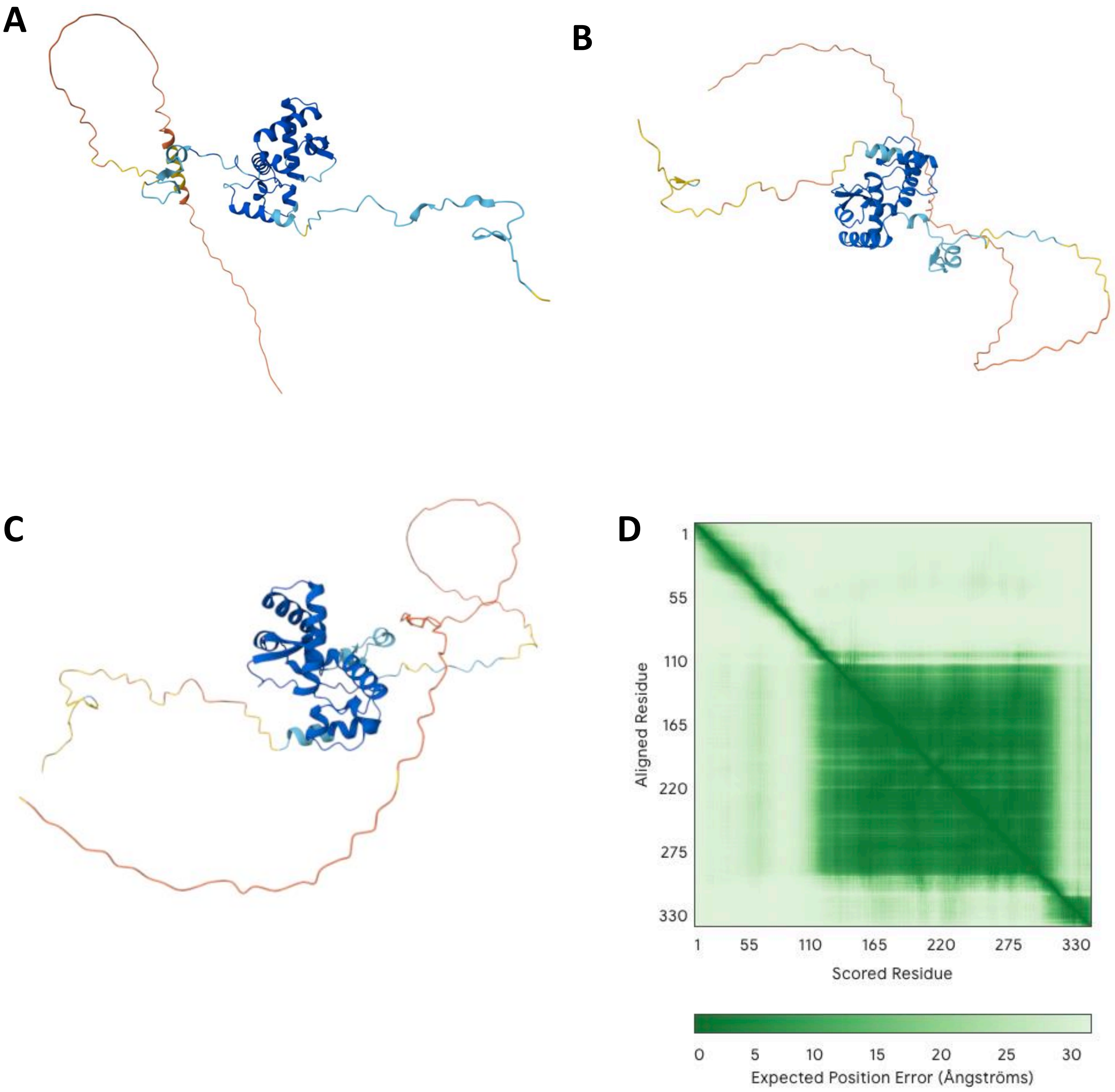

Supplementary Figure 9. Unphosphorylated monomer coat protein (CP) structure predicted by AlphaFold3. (A) upper view; (B) side view; (C) bottom view; (D) alignment vs. score plot.
