# Supplementary material for: Structural characterization of plum pox virus by cryo-electron microscopy
Source: Arch Virol. 2025 Dec 1;171(1):11. doi: 10.1007/s00705-025-06473-5 (PMC12669337; doi:10.1007/s00705-025-06473-5)
Supplement: Supplementary file 17 — Supplementary Material 17 (PDF 229 KB) [file 705_2025_6473_MOESM17_ESM.pdf]

Structural characterization of plum pox virus (PPV) by cryo-EM
Archives of Virology
Diane Marie Valérie Jeanne Bonnet, Antonio Chaves-Sanjuan, Nicoletta Contaldo, Angelo De Stradis, Rosanna Caliandro, Angelantonio Minafra, Filippo Geuna*

***Corresponding author: filippo.geuna@unimi.it**
Department of Agricultural and Environmental Sciences (DISAA) - Università degli Studi di Milano, Milan, Italy

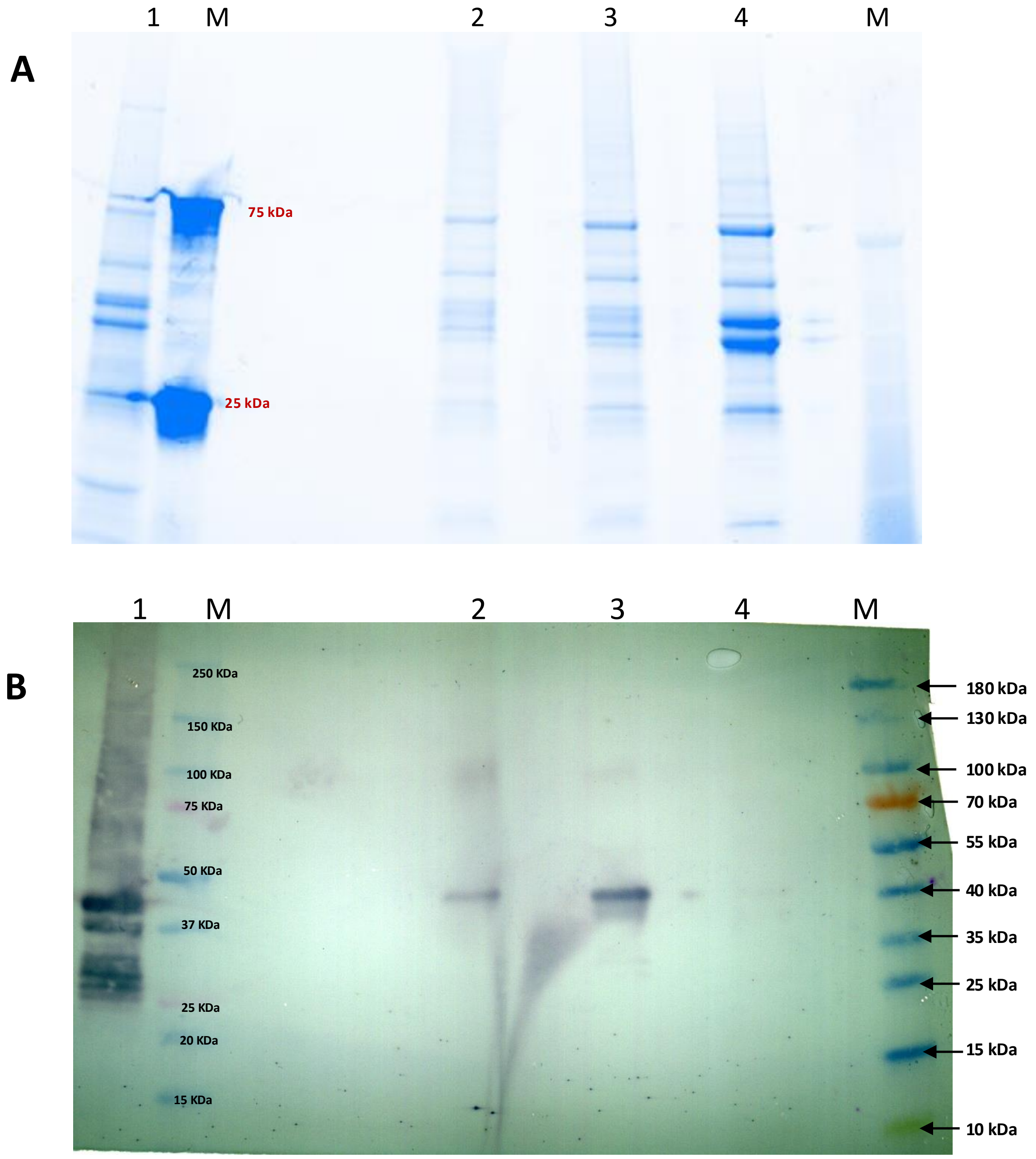

Supplementary Figure 11. SDS-PAGE and Western blot analysis of the PPV coat protein. (A) An SDS-PAGE was stained by Coomassie brilliant blue and (B) Western blot analysis on a twin gel was performed using a partially purified virus aliquot (lane 1), in comparison with denatured PBS extracts of healthy (lane 4) and PPV-infected *N. benthamiana* plants (lane 2, supernatant; lane 3, pellet). The PVDF blotted membrane was probed with a 1:1000-diluted commercial polyclonal antibody preparation (AP-conjugated; Agritest). Marker ladders are indicated with their molecular weights.
