# Supplementary material for: Structural characterization of plum pox virus by cryo-electron microscopy
Source: Arch Virol. 2025 Dec 1;171(1):11. doi: 10.1007/s00705-025-06473-5 (PMC12669337; doi:10.1007/s00705-025-06473-5)
Supplement: Supplementary file 12 — Supplementary Material 12 (PDF 329 KB) [file 705_2025_6473_MOESM12_ESM.pdf]

Structural characterization of plum pox virus (PPV) by cryo-EM
Archives of Virology
Diane Marie Valérie Jeanne Bonnet, Antonio Chaves-Sanjuan, Nicoletta Contaldo, Angelo De Stradis, Rosanna Caliandro, Angelantonio Minafra, Filippo Geuna*

***Corresponding author: filippo.geuna@unimi.it**
Department of Agricultural and Environmental Sciences (DISAA) - Università degli Studi di Milano, Milan, Italy

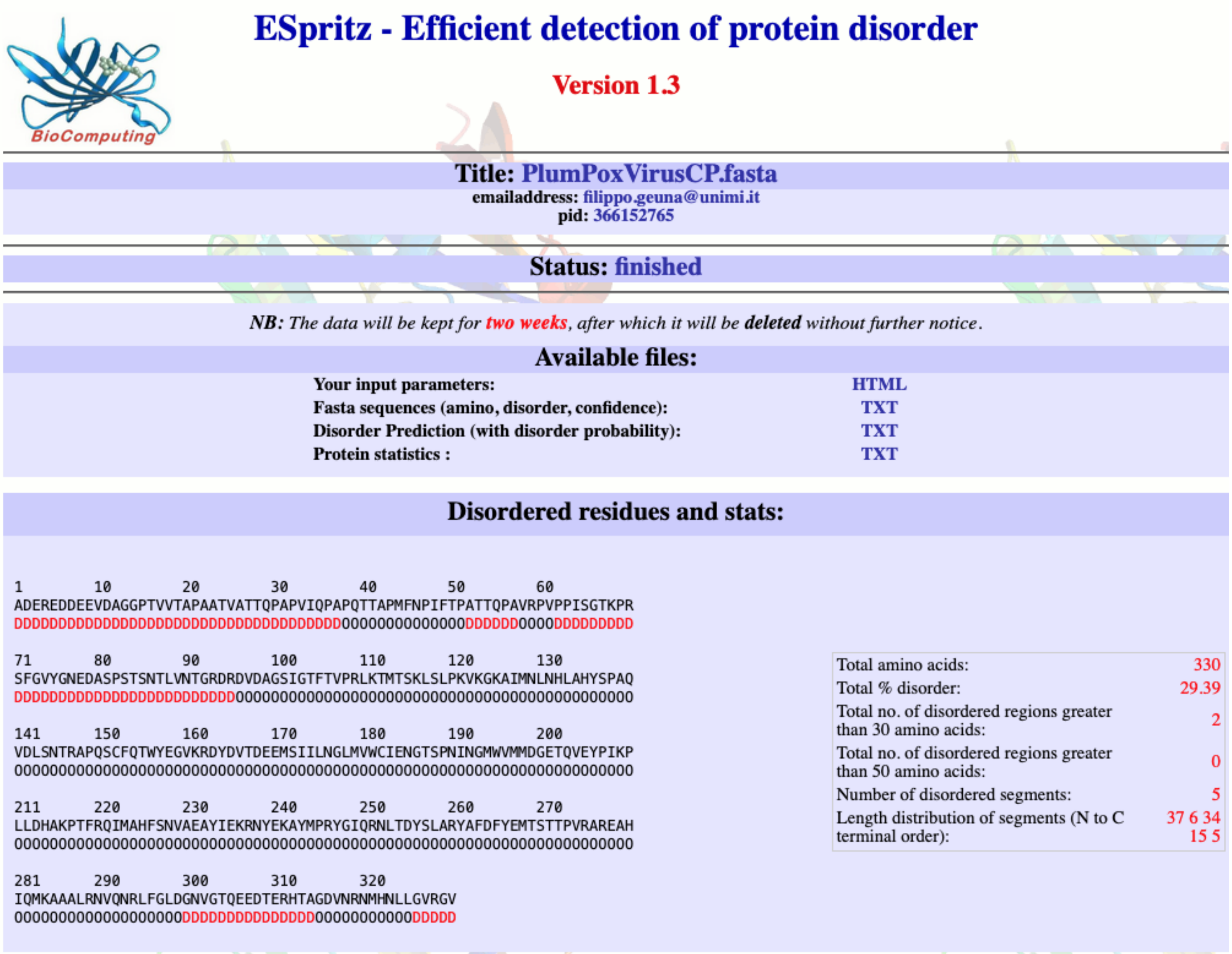

Supplementary Figure 6. *In silico* prediction of disordered regions of CP. The disordered segments with their extensions are reported (disordered amino acid residues are marked by red 'D's below the protein sequence) along with those greater than 30 amino acids.
